# Impact of CFTR Modulation on *Pseudomonas aeruginosa* Infection in People With Cystic Fibrosis

**DOI:** 10.1093/infdis/jiae051

**Published:** 2024-03-05

**Authors:** Emma L Ledger, Daniel J Smith, Jing Jie Teh, Michelle E Wood, Page E Whibley, Mark Morrison, Joanna B Goldberg, David W Reid, Timothy J Wells

**Affiliations:** Frazer Institute, Faculty of Medicine, The University of Queensland, Brisbane, Australia; Northside Clinical Unit, The University of Queensland, Brisbane, Australia; Adult Cystic Fibrosis Centre, The Prince Charles Hospital, Brisbane, Australia; Frazer Institute, Faculty of Medicine, The University of Queensland, Brisbane, Australia; Adult Cystic Fibrosis Centre, The Prince Charles Hospital, Brisbane, Australia; Adult Cystic Fibrosis Centre, The Prince Charles Hospital, Brisbane, Australia; Frazer Institute, Faculty of Medicine, The University of Queensland, Brisbane, Australia; Australian Infectious Diseases Research Centre, Brisbane, Australia; Department of Pediatrics, Division of Pulmonary, Asthma, Cystic Fibrosis, and Sleep, Emory University School of Medicine, Atlanta, Georgia, USA; Northside Clinical Unit, The University of Queensland, Brisbane, Australia; Australian Infectious Diseases Research Centre, Brisbane, Australia; QIMR Berghofer Medical Research Institute, Brisbane, Australia; Frazer Institute, Faculty of Medicine, The University of Queensland, Brisbane, Australia; Australian Infectious Diseases Research Centre, Brisbane, Australia

**Keywords:** CFTR modulators, cystic fibrosis, elexacaftor-tezacaftor-ivacaftor, ETI, *Pseudomonas aeruginosa*

## Abstract

**Background:**

*Pseudomonas aeruginosa* is a multidrug-resistant pathogen causing recalcitrant pulmonary infections in people with cystic fibrosis (pwCF). Cystic fibrosis transmembrane conductance regulator (CFTR) modulators have been developed that partially correct the defective chloride channel driving disease. Despite the many clinical benefits, studies in adults have demonstrated that while *P. aeruginosa* sputum load decreases, chronic infection persists. Here, we investigate how *P. aeruginosa* in pwCF may change in the altered lung environment after CFTR modulation.

**Methods:**

*P. aeruginosa* strains (n = 105) were isolated from the sputum of 11 chronically colonized pwCF at baseline and up to 21 months posttreatment with elexacaftor-tezacaftor-ivacaftor or tezacaftor-ivacaftor. Phenotypic characterization and comparative genomics were performed.

**Results:**

Clonal lineages of *P. aeruginosa* persisted after therapy, with no evidence of displacement by alternative strains. We identified commonly mutated genes among patient isolates that may be positively selected for in the CFTR-modulated lung. However, classic chronic *P. aeruginosa* phenotypes such as mucoid morphology were sustained, and isolates remained just as resistant to clinically relevant antibiotics.

**Conclusions:**

Despite the clinical benefits of CFTR modulators, clonal lineages of *P. aeruginosa* persist that may prove just as difficult to manage in the future, especially in pwCF with advanced lung disease.

## Teaser

We found that clonal strains of *Pseudomonas* persist after elexacaftor-tezacaftor-ivacaftor treatment with the same “chronic” phenotypes that are just as clinically challenging in people with cystic fibrosis. We further identified commonly mutated bacterial genes that may drive future adaptation to the CFTR (cystic fibrosis transmembrane conductance regulator) modulated lung.

Cystic fibrosis (CF) is caused by mutations in the *CFTR* gene (CF transmembrane conductance regulator) that result in defective cellular chloride transport [1]. CF is a multisystem disorder, although the predominant cause of morbidity and mortality is due to severe progressive pulmonary disease as a consequence of recurrent and chronic respiratory infections [1].

A principal opportunistic pathogen causing recalcitrant pulmonary infections in people with CF (pwCF) is *Pseudomonas aeruginosa*. This gram-negative and highly multidrug-resistant bacterium can adapt to the hostile CF lung environment to establish persistent infections that are extremely difficult to eradicate [2]. Development of chronic infection is characterized by the downregulation of bacterial virulence factors, such as oligosaccharide antigen (O-antigen), and the formation of mucoid colonies that overproduce the exopolysaccharide alginate that provides stability for the formation of biofilms [2, 3]. Established clonal communities often persist and continue to diversify for decades [2, 4]; however, highly transmissible *P. aeruginosa* strains can displace existing chronic communities or co-colonize pwCF, causing mixed-strain infections that further complicate treatment regimens [4–7]. *P. aeruginosa* infections are correlated with increased pulmonary exacerbations, accelerated lung function decline, impaired quality of life, and early death in pwCF [8].

The advent of effective CFTR modulators in CF has radically changed the landscape of CF health care. The latest triple-combination therapy, elexacaftor-tezacaftor-ivacaftor (ETI; Trikafta), has been found to have the greatest clinical benefits, with up to 90% of pwCF having a genotype responsive to this therapy [9, 10]. Demonstrated benefits of CFTR modulator therapy in pwCF include improvement in lung function and nutritional health indices, a reduced incidence of pulmonary exacerbations, and improved quality of life [10, 11].

Despite the clear benefits to health in pwCF, the impact of CFTR modulators on chronic *P. aeruginosa* airway infection remains undercharacterized. Observational studies show a reduction in *P. aeruginosa*–positive sputum cultures by up to 55% in the first year of CFTR modulator treatment [12–14], but this apparent eradication was mainly in individuals who were intermittently colonized. In chronic *P. aeruginosa* infection, modulators have been associated with a reduction in *P. aeruginosa* bacterial load but not complete eradication [15–17].

In this study, we aimed to characterize the changes in genotype and phenotype of persisting *P. aeruginosa* strains collected for up to 21 months after CFTR modulation in pwCF receiving mainly ETI or tezacaftor-ivacaftor (TI). Understanding how CFTR modulators affect CF respiratory infections is critical to direct future research and clinical care, especially for *P. aeruginosa*, which is notoriously difficult to treat.

## METHODS

### Participant Recruitment

**Figure 1.**
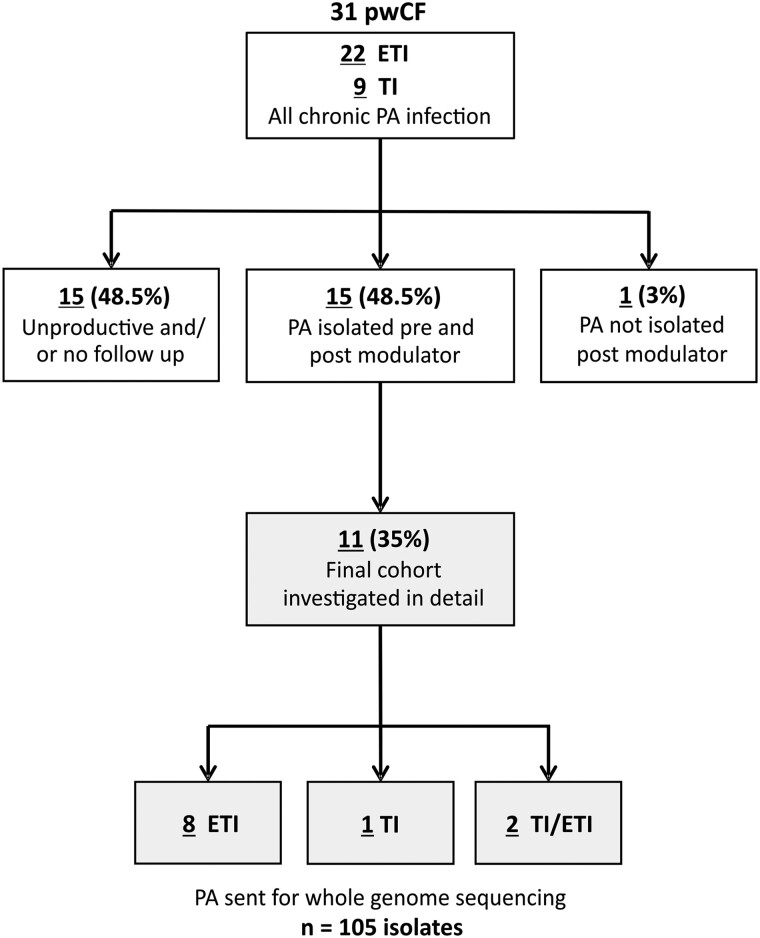
Workflow of participant recruitment, cystic fibrosis transmembrane conductance regulator (CFTR) modulator therapy, and *Pseudomonas aeruginosa* (PA) collection. Thirty-one people with cystic fibrosis (pwCF) were initially recruited. TI/ETI indicates that isolates were collected prior to participants starting tezacaftor-ivacaftor (TI) before switching to elexacaftor-tezacaftor-ivacaftor (ETI).

Thirty-one adults with CF and chronic *P. aeruginosa* infection commencing TI (n = 9) or compassionate access ETI (n = 22) were recruited between September 2019 and October 2020 after human research and ethics committee (HREC/2019/QPCH/46169) approval and participant written consent (Figure 1). Chronic infection was defined as a participant having *P. aeruginosa* present in >50% of the last six sputum cultures collected over a minimum of 12 months [18]. Fifteen patients were not able to provide follow-up samples due to a combination of becoming unproductive for sputum following commencement of therapy and/or failure to attend face-to-face follow-up due to the coronavirus pandemic response. This led our study to include pwCF with more severe lung disease and a smaller sample size than desired. One participant, although productive, became culture negative for *P. aeruginosa* and therefore could not be studied. Fifteen participants provided spontaneously expectorated sputum samples before and at various time points up to 21 months after CFTR modulator commencement. Of these 15 patients, a final cohort of 11 pwCF receiving ETI (n = 8), TI (n = 1), and TI followed by ETI (n = 2) were investigated in detail (Figure 1). Participants one to 11 are labeled with the code CF participant (CFP) throughout the study.

### Clinical Data Collection

Clinical details of the 11 participants were collected for the 12 months prior to and 12 months after modulator commencement. Data collected included best forced expiratory volume in 1 second percent predicted (FEV1pp; calculated with Global Lung Function Initiative standards), body mass index (BMI), and number of exacerbations/intravenous antibiotic use. Sweat chloride was collected with the Macroduct sweat collection system per the manufacturer's instructions at the time of therapy commencement and between 1 and 12 months posttreatment.

### Bacterial Isolation and Culture

A small plug of sputum was inoculated onto horse blood Columbia agar (PP2001; Thermo Fisher Scientific) and MacConkey agar (base 3, CM0115; OXOID). Plates were incubated for up to 72 hours at 37 °C ± 5% CO_2_. Morphologically distinct colonies were selected, grown in lysogeny broth at 37 °C, shaken at 220 revolutions per minute overnight, and stored in 20% glycerol at −80 °C. Bacterial species were confirmed by MALDI-TOF (matrix-assisted laser desorption/ionization–time of flight) VITEK mass spectrometry.

### Whole Genome Sequencing

All 105 *P. aeruginosa* isolates were cultured in lysogeny broth by overnight shaking, and genomic DNA was extracted with the Qiagen DNA Mini Kit per the manufacturer's instructions. Genomic DNA was sent to Microbes NG (https://microbesng.com) for Illumina sequencing with 2 × 250–bp paired-end reads with a minimum 30× depth coverage. See supplementary data 1 for methods on genome assembly and quality assessment.

### Comparative Genomics

Genome sequences were queried in PubMLST.org to determine multilocus sequence type (MLST) profiles that differentiated strains by sequence variation (alleles) of seven housekeeping genes [19]. Sequence types that are close together in numerical value do not indicate closely related strains. Assembled contigs were queried with PAst V1.0, classifying *P. aeruginosa* isolates into one of 11 serogroups based on BLAST analysis of the O-specific antigen gene cluster (covering the 20 International Antigenic Typing Scheme serotypes) [20]. We utilized Snippy 4.6.0 (https://github.com/tseemann/snippy) to call variants, including single-nucleotide polymorphisms and indel mutations, from the whole genome shotgun sequences of 90 isolates from persisting clonal lineages (14 clonal lineages from 11 patients) against the *P. aeruginosa* PAO1 reference genome (assembly accession GCF_000006765.1 [21]). See the supplementary data 1 for detailed methods of the supplementary figures and variant calling performed. Figures were created in R Studio with the *ggplot* and *pheatmap* packages and BioRender.com.

### O-antigen Expression

O-antigen expression was determined through sodium dodecyl sulfate–polyacrylamide gel electrophoresis of patient isolate lipopolysaccharide and by Western blotting with polyvalent *P. aeruginosa* O-antigen–specific antiserum (200372; Denka Company Limited). See supplementary data 1 for detailed methods.

### Antibiotic Susceptibility Testing

To determine antibiotic susceptibility profiles of *P. aeruginosa* isolates, disc diffusion was performed per the Clinical Laboratory Standards Institute guidelines and standards. See supplementary data 1 for detailed methods.

### Statistical Analyses

All statistical analyses were performed in Prism (version 9; GraphPad). A Wilcoxon signed rank paired test was performed for comparing clinical parameter outcomes, an unpaired Mann-Whitney test for hypermutator isolate analysis, and a Fisher's exact test for pathoadaptive and antibiotic gene enrichment and for comparison of number of isolates with chronic phenotypes (O-antigen expression, mucoid morphology, antibiotic susceptibility). *P* < .05 was considered significant.

## Supplementary Material

jiae051_Supplementary_Data

## Figures and Tables

**Table 1.** Cohort Characteristics at Baseline

	No. (%) or Median (IQR)			
Characteristic	Whole Cohort	ETI	TI	TI/ETI^a^
Patients	11	8	1	2
F508del homozygous genotype	6 (55)	4	1	1
F508del heterozygous genotype	5 (45)	4	…	1
Age, y	30.1 (26.7–32.1)	30.3 (28.1–31.8)	26	36 (28.8–43.2)
Female:male	5:6	4:4	0:1	1:1
Best FEV1pp 12 mo prior	39.8 (35.9–42.9)	41.7 (37.1–43.3)	39	32.3 (28.6–36.0)
Best BMI 12 mo prior, kg/m^2^	20.8 (19.7–23.6)	20.2 (19–21.5)	37	29.5 (25.9–33.0)
Sweat chloride, mmol/L^b^	95 (85–100)	97 (87–100.5)	85	90
No. of exacerbations 12 mo prior	3 (2–4)	3 (2–4)	1	4 (3–5)
Previous CFTR modulator use	5 (45)	4	1	…
No. of patients with pulmonary colonization				
Mucoid *P. aeruginosa*^c^	5	5	…	…
Nonmucoid *P. aeruginosa*	10	7	1	2
*Staphylococcus aureus*	1	…	…	

Abbreviations: BMI, body mass index; ETI, elexacaftor-tezacaftor-ivacaftor; FEV1pp, forced expiratory volume in 1 second percent predicted; *P. aeruginosa*, *Pseudomonas aeruginosa*; TI, tezacaftor-ivacaftor.

^a^Baseline characteristics are prior to naive TI commencement.

^b^Baseline sweat chloride was not available for all patients (n = 9).

^c^Two additional patients had cultured mucoid *P. aeruginosa* in previous recent sputum samples prior to study isolation (not included in table).

**Table 2.** Median Changes in Clinical Parameters From the 12 Months Prior to the 12 Months After CFTR Modulator Therapy in People With Cystic Fibrosis

	Best FEV1pp in 12 mo			Best BMI in 12 mo, kg/m^2^			Sweat Chloride, mmol/L (n = 9)^a^			No. of Exacerbations in 12 mo		
	Prior	After	Median Change	Prior	After	Median Change	Baseline	After	Median Change	Prior	After	Median Change
All ETI (n = 10)^b^												
Median	40.7	46.4	5.1	20.5	22.7	1.1	96.0	44.5	–51.5	3.5	.0	–2.5
IQR	34.8–43.0	42.0–47.7	2.3–6.2	19.4–23.0	20.6–23.8	0.4–2.1	86.5–99.5	37.8–56.8	–56.3 to –18.8	2.2–4.8	0–0	–4 to –1.3
*P* value			**.0098**			.**0391**			.**0078**			.**0039**
Naive-ETI (n = 4)												
Median	41.1	46.4	2.5	21.9	23.1	1.8	95.0	40.0	–53.0	2.5	0.0	–2.0
IQR	37.1–44.6	44.9–46.7	1.7–5.5	19.1 –23.6	21.5–24.3	1.2–2.5	87–96	33–57	–55 to –37	2–3.3	0–0.8	–2.5 to –1.5
*P* value			.125			.125			.25			.25
LI/TI-ETI (n = 6)^b^												
Median	40.7	45.9	6.0	20.5	22.0	0.6	99.0	46.0	–50.0	4.5	0.0	–3.5
IQR	32.4–42.3	38.2–48.2	4.7–6.2	19.4–21.2	20.6–23.3	0.1–1.7	89–101	43–51	–56 to –12	3.3–5	0–0	–4.75 to –1.5
*P* value			.0625			.3125			.0625			.**0312**
All TI (n = 3)												
Median	39.3	41.7	2.5	36.5	36.2	–0.4	90.0	89.0	–1.0	2.0	3.0	–1.0
IQR	32.1–39.5	31.2–42.2	–0.9 to 2.7	29.5–36.8	28.8–36.4	–0.7 to −0.4	90–90	89–89	–1 to –1	1.5–4	1.5–4	2 to –1
*P* value			NT			NT			NT			NT

A Wilcoxon matched-pairs signed rank test was performed comparing clinical parameters before and after CFTR modulator commencement. Bold values indicate *P* < .05.

Abbreviations: BMI, body mass index; CFP, cystic fibrosis participant; CFTR, cystic fibrosis transmembrane conductance regulator; ETI, elexacaftor-tezacaftor-ivacaftor; FEV1pp, forced expiratory volume in 1 second percent predicted; LI, lumacaftor-ivacaftor; NT, no test performed due to small sample size; TI, tezacaftor-ivacaftor.

^a^Either no sweat chloride data at baseline or no matched postmeasurements were available for four patients (CFP 4, 6, 10, and 11).

^b^Two patients started the study taking TI and later transitioned to ETI; therefore, their clinical parameters were represented twice and included in both the ETI and TI groups accordingly.

**Figure 2.**
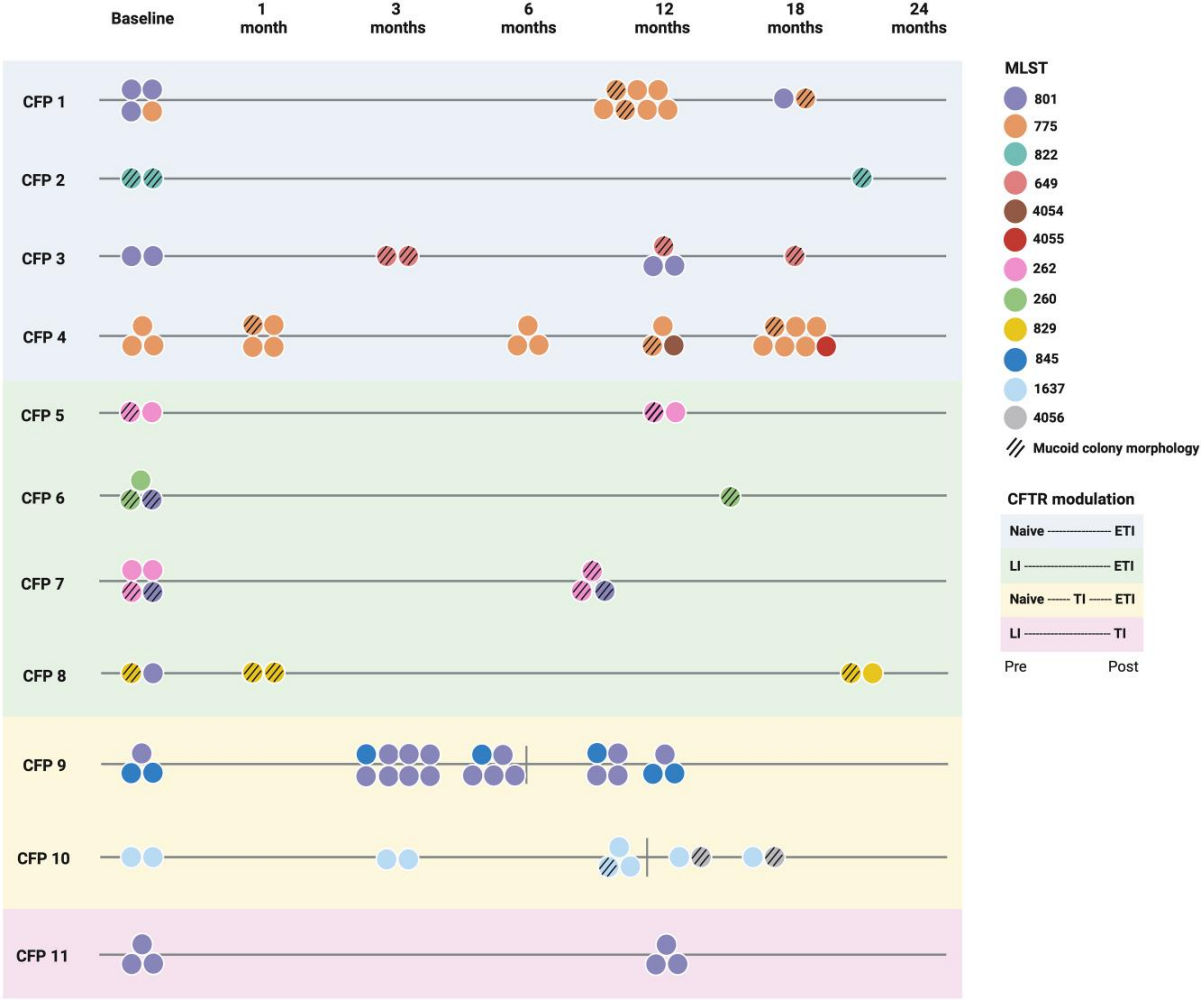
Clonal *Pseudomonas aeruginosa* sequence types persist up to 21 months after CFTR modulator commencement in people with cystic fibrosis. Bacteria were isolated at baseline and various time points after CFTR modulator initiation. Each bacterial strain isolated is represented by a circle, and the color indicates the multilocus sequence type (MLST). Circles with three intersecting lines indicate that the isolate was of mucoid morphology. Shaded background indicates the CFTR modulator therapy characteristics. Cystic fibrosis participants (CFP 9 and 10) who commenced tezacaftor-ivacaftor (TI) and then switched to elexacaftor-tezacaftor-ivacaftor (ETI) are indicated by the line intersecting the x-axis at approximately six and 12 months. Figure created in BioRender. CFTR, cystic fibrosis transmembrane conductance regulator; LI, lumacaftor-ivacaftor.

**Table 3.** Mutations Accumulated in Lineages After CFTR Modulation in People With Cystic Fibrosis

		No. Isolates Analyzed		Mutations Unique to Isolates Post–CFTR Modulation^a^			No. of Mutated Genes				No. of Mutated Genes		
Patient_MLST	Time Since Modulation, mo	Baseline	After	Total	SNP	Indel	Unique genes	Pathoadaptive (n = 159)	Other	*P* Value	Antibiotic Resistant (n = 185)	Other	*P* Value
CFP1_801	18	3	1	90	69	21	72	4	68	.1398	3	69	.5062
CFP1_775^b^	18	1	5	418	276	142	354	50	304	**<**.**0001**	26	328	**<**.**0001**
CFP2_822	21	2	1	202	142	60	190	9	181	.1104	6	184	>.9999
CFP3_801	16	2	2	92	59	33	84	10	74	**<**.**0001**	2	82	>.9999
CFP4_775	17	3	14	3400	2652	748	2176	100	2076	**<**.**0001**	85	2091	.**0309**
CFP5_262	14	2	2	82	49	33	72	7	65	.**0037**	1	71	.7310
CFP6_260	16	2	1	19	11	8	18	2	16	.0880	0	18	>.9999
CFP7_262	8	3	1	183	148	35	178	15	163	.**0001**	6	172	.8294
CFP7_801	8	1	2	1042	816	226	629	42	587	**<**.**0001**	30	599	.**0305**
CFP8_829	20	1	4	664	437	227	568	55	513	**<**.**0001**	26	542	.0611
CFP9_845	13	2	5	1711	1342	369	1282	72	1210	**<**.**0001**	54	1228	.**0311**
CFP9_801	13	1	15	522	357	165	444	30	414	**<**.**0001**	20	424	.1234
CFP10_1637	15	2	6	70	27	43	54	6	48	.**0036**	3	51	.2541
CFP11_801	12	3	3	122	86	36	112	11	101	.**0003**	4	108	.7844

*P* values represent a Fisher's exact test of the number of mutated pathoadaptive genes or antibiotic-resistant genes (Supplementary Tables 5 and 6) in each lineage versus other genes mutated within the genome. Bold values indicate *P* < .05.

Abbreviations: CFP, cystic fibrosis participant; CFTR, cystic fibrosis transmembrane conductance regulator; MLST, multilocus sequence type; SNP, single-nucleotide polymorphism.

^a^Nonsynonymous and modifier indel or frameshift mutations.

^b^This lineage includes MLSTs 775, 4054, and 4055.

**Figure 3.**
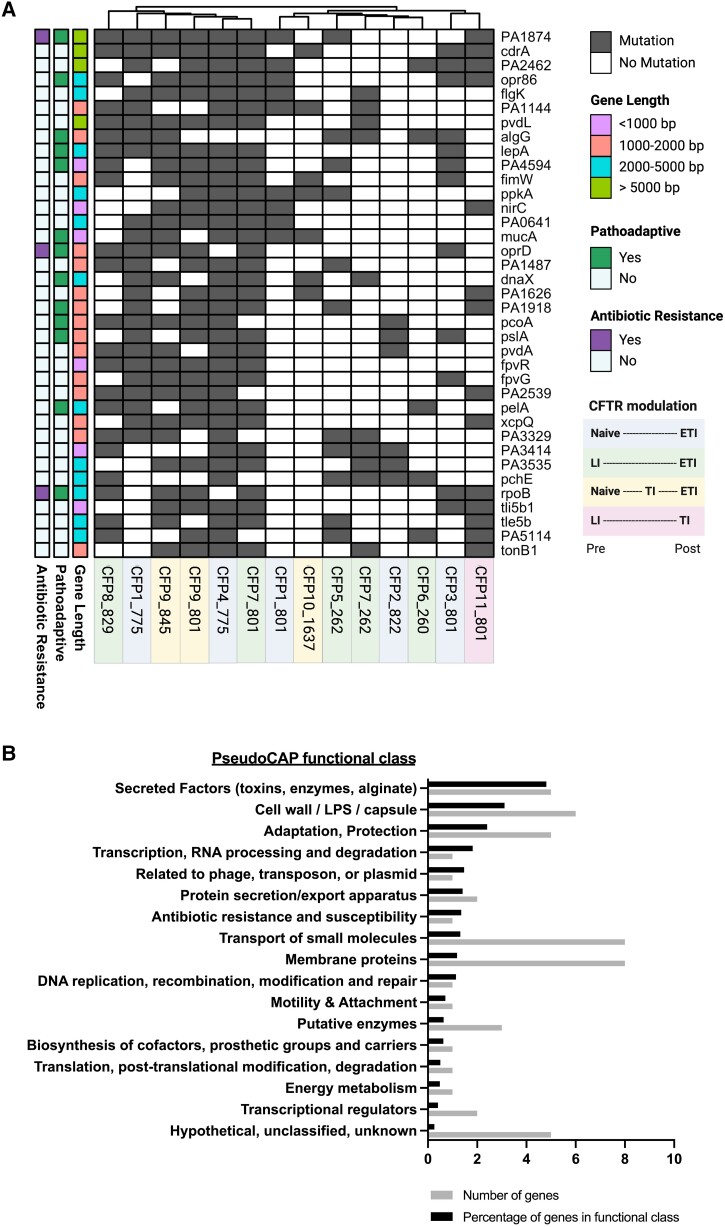
Genes mutated after CFTR modulation in >42% of lineages from people with cystic fibrosis. *A*, Presence-and-absence heat map of 37 commonly mutated genes (≥6/14, 42%) with at least one nonsynonymous modifier indel or frameshift mutation accumulated in lineages after CFTR modulation in people with cystic fibrosis. Heat map is annotated with gene length and whether the gene was previously described as being pathoadaptive or involved in antibiotic resistance. Lineages are shaded by CFTR modulator therapy characteristics. *B*, PseudoCAP functional class of 37 commonly mutated genes (number and percentage of genes relative to number of genes in class). Figure 3*A* created in R Studio and BioRender. CFTR, cystic fibrosis transmembrane conductance regulator; ETI, elexacaftor-tezacaftor-ivacaftor; LI, lumacaftor-ivacaftor; LPS, lipopolysaccharide; TI, tezacaftor-ivacaftor.

**Figure 4.**
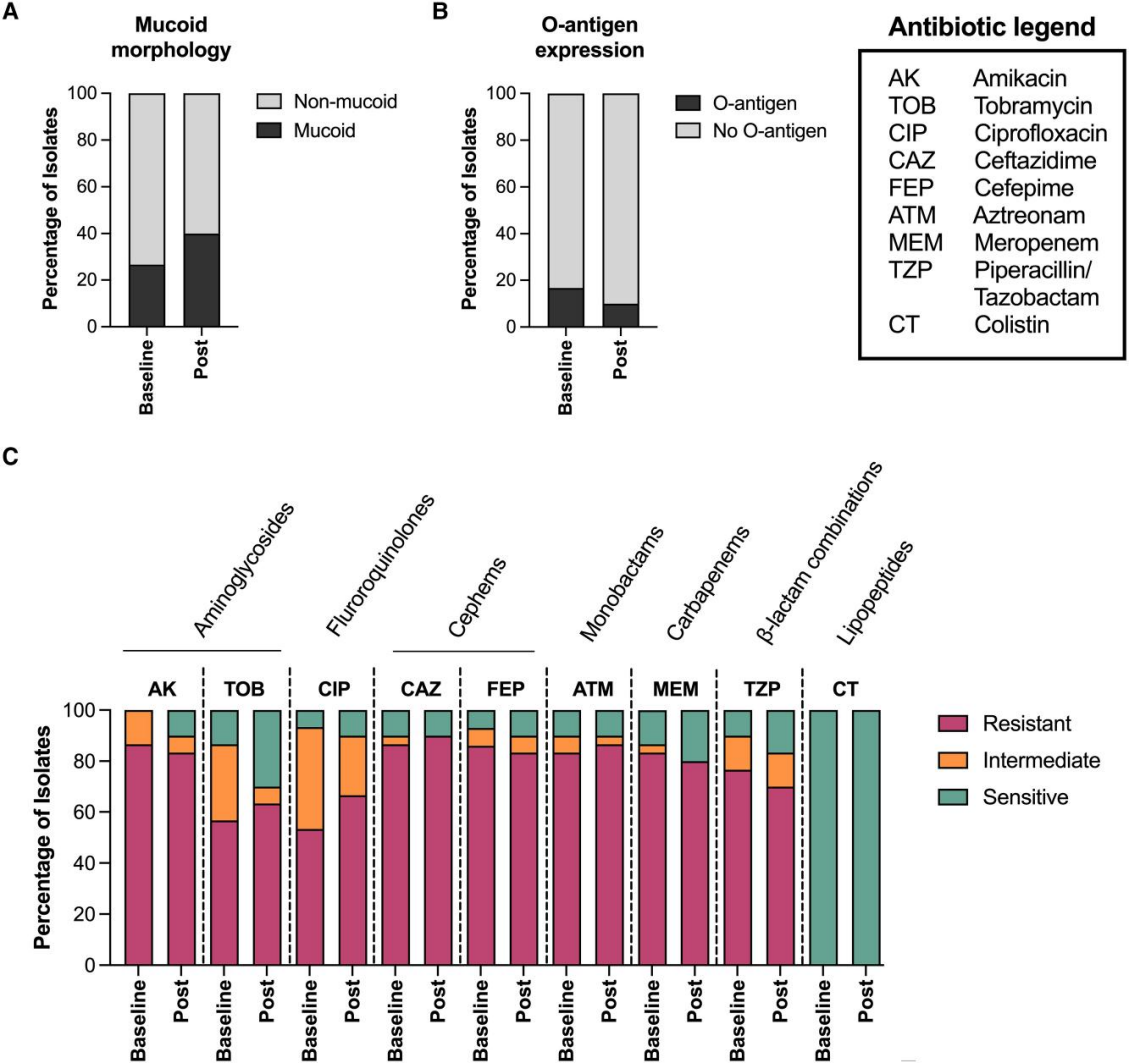
Chronic *Pseudomonas aeruginosa* phenotypes are sustained after CFTR modulation in people with cystic fibrosis. *P. aeruginosa* (*A*) mucoid colony morphology, (*B*) O-antigen expression, and (*C*) antibiotic resistance at baseline and the latest time point available after CFTR modulator therapy. *Resistant*, resistant to antibiotics; *intermediate*, sensitive, although increased antibiotic dosing is advised; *sensitive*, completely sensitive to antibiotics. No significant differences were found. CFTR, cystic fibrosis transmembrane conductance regulator.

## Data Availability

All *P. aeruginosa* genomes are available on NCBI BioProject PRJNA1023362 (see Supplementary Table 1 for corresponding BioSample accession numbers). All isolate phenotypic data are available in Supplementary Table 2, clinical data in Supplementary Table 3, and all filtered mutations in Supplementary Data 2 and 3.

## RESULTS

### Cohort Characteristics

Of the 11 participants who provided multiple *P. aeruginosa*–positive samples, each had at least one copy of the F508del *CFTR* mutation. The cohort had a median age of 30.1 years and an almost equal proportion of males to females (Table 1). Eight patients received ETI, one TI, and two initially commenced TI but then switched to ETI six and 12 months after commencement (Figure 1). Almost half (45%) were utilizing lumacaftor-ivacaftor at the beginning of the study. At baseline, the median sweat chloride of 95 mmol/L was above diagnostic levels for CF (>60 mmol/L). The median best FEV1pp in 12 months prior to commencement was 40%, reflecting a cohort of participants with advanced lung disease. A total of 105 *P. aeruginosa* isolates were collected. Only 1 patient was co-colonized with another CF pathogen, *Staphylococcus aureus*, prior to modulator commencement.

### Clinical Improvements After CFTR Modulation

Clinical outcomes in the 12 months prior to and 12 months after CFTR modulation were compared. In patients receiving ETI, there was a median 5.1% increase in lung function (FEV1pp; Table 2, Supplementary Figure 1*A*). The median BMI increased from 20.5 to 22.7 kg/m^2^ (Supplementary Figure 1*B*). Median sweat chloride fell by 51.5 mmol/L, with all except three patients dropping below the diagnostic level for CF (>60 mmol/L; Supplementary Figure 1*C*), and the median number of pulmonary exacerbations decreased from three to zero (Supplementary Figure 1*D*). Similar improvements in clinical parameters were seen in people who transitioned to ETI from an alternate modulator as compared with those commencing ETI for the first time. Of three participants receiving TI, two had a 2.5% and 2.9% increase in FEV1pp but a decrease in BMI and variable change in pulmonary exacerbations. One TI participant (CFP9; age, 50 years) deteriorated throughout the study duration. Overall, we observed that all except one participant had significant improvements across multiple clinical parameters after CFTR modulation.

### Clonal *P. aeruginosa* Persists After CFTR Commencement

To determine whether participants were colonized with the same clonal *P. aeruginosa* lineages after CFTR modulation, we sequenced all 105 *P. aeruginosa* strains and compared their sequence similarity and MLST profile that differentiated strains based on variation within the housekeeping genes to determine clonality before and after treatment. At baseline, six participants were colonized with a single MLST while five participants were colonized with two *P. aeruginosa* sequence types (Figure 2). *P. aeruginosa* MLST 801 was shared among seven participants and MLST 775 between two participants, which both corresponded to the profile of two endemic strains (AUST-O6, AUST-O2) circulating in Australian CF centers [5, 6]. Sequence types 649 (AUST-O1), 822 (AUST-11), and 262 (AUST-O7) have also been isolated frequently from the same CF centre as this study [6].

For all participants, at least one of the *P. aeruginosa* lineages with which they were initially colonized persisted throughout the entire study duration, as reflected in MLST and phylogeny (Figure 2, Supplementary Figure 2). Only two patients (CFP 6 and CFP 8) had a sequence type isolated at baseline (MLST 801, AUST-O6) that was not isolated again after CFTR modulator therapy. New sequence types were isolated from two participants (CFP 4 and CFP 10) after 12 months of therapy. The two sequence types isolated posttreatment from CFP 4 (MLST 4054 and 4055) had the same central genotype that differed in variation at only two of seven loci compared with MLST 775 isolated at baseline, and therefore clustered in the same clonal complex (Supplementary Figures 2 and 3). In contrast, the new sequence type from CFP 10 (MLST 4056) did not cluster with other baseline strains and had a unique O-antigen serotype representing a novel strain.

### Genetic Adaption of *P. aeruginosa* After CFTR Modulation

Next, we wanted to determine any indication of adaptation to the new modulated lung environment by identifying mutations unique to clonal strains isolated after CFTR modulation. Fourteen clonal lineages from 11 participants persisted after CFTR modulation and accumulated 19 to 3400 mutations (nonsynonymous, modifier microindel, or frameshift) within 3565 genes in the PAO1 reference genome (Table 3). In 11 of 14 lineages, mutations were more frequent in previously described pathoadaptive genes identified in longitudinal *P. aeruginosa* isolates in pwCF [2, 4, 22–26]. In contrast, we observed only four lineages having mutations occurring more often in genes involved in antibiotic resistance [27–29].

To determine if the new lung environment was selecting for mutations in specific genes, we identified mutations in genes shared across a large proportion of lineages. We found that 37 genes were mutated in >42% (6/14) of lineages (Figure 3*A*, Supplementary Table 4). Of these genes, 13 (35%) were previously described as pathoadaptive and three were associated with antibiotic resistance. Lineages that clustered closely in number of genes commonly mutated accumulated a large amount of overall mutations (>400) after CFTR modulation and had significantly more mutations in *mutS*, *mutL*, and *urvD* genes responsible for DNA mismatch repair, which have been shown to cause hypermutation [30]. The two genes mutated in the most lineages (9/14, 64%) were *PA1874*, which encodes for a hypothetical protein previously described as pathoadaptive, and *cdrA*, an extracellular matrix protein [31]. The functional class of “secreted factors (toxins, enzymes, alginate)” had the highest frequency of mutated genes relative to the number of genes in the corresponding class, including *algG*, *lepA*, *pchE*, *cdrA*, and *tle5b* [31] (Figure 3*B*). Other classes with high frequency of mutated genes were “cell wall/lipopolysaccharide/capsule” and “adaption, protection” which contained additional commonly mutated genes, such as *mucA*, *flgK*, *pslA*, *pelA*, *pcoA*, *ppkA*, *pvdA*, and *pvdL*. As all these genes were mutated in multiple lineages, they may represent selection of mutations driven by the new modulated lung environment.

### Chronic *P. aeruginosa* Phenotypes Are Sustained

Last, we wanted to determine whether classically chronic *P. aeruginosa* phenotypes were sustained after CFTR modulation. To do this, baseline isolate characteristics were compared with isolates from the latest time point for each participant. There were no significant changes observed in the proportion of isolates with mucoid morphology (Figure 2), O-antigen expression, and antibiotic resistance (Figure 4). Slight changes were due to small differences in the number of strains isolated per participant at each time point. Between 57% and 90% of isolates were resistant to antibiotics routinely used to treat this cohort of participants, including tobramycin, ceftazidime, aztreonam, meropenem, cefepime, and piperacillin-tazobactam. Five participants were colonized with at least 1 isolate that was resistant to all antibiotics tested except colistin. Thus, pwCF remain colonized with clonal lineages of *P. aeruginosa* with the same chronic phenotypes that are notoriously difficult to manage clinically.

## DISCUSSION

*P. aeruginosa* is a problematic pathogen associated with increased morbidity and mortality in pwCF [32]. While previous studies have demonstrated that CFTR modulators can drive decreases in *P. aeruginosa* bacterial load in sputum, complete eradication is not achieved [15, 16]. In our cohort of 11 pwCF with advanced lung disease and chronic *P. aeruginosa* infection, we demonstrated that despite improvements in health, the same clonal *P. aeruginosa* isolates persisted up to 21 months after ETI or TI commencement. Mutations unique to strains isolated after CFTR modulation were more often in previously described pathoadaptive genes in 11 of 14 lineages, while only four lineages accumulated mutations more frequently in antibiotic-resistant genes. Additionally, 37 genes were identified to be mutated across a sizable proportion of lineages; however, chronic *P. aeruginosa* phenotypes were sustained, such as mucoid morphology, lack of O-antigen expression, and antibiotic resistance.

Multiple clinical improvements were observed in our study, although increases in lung function were modest as compared with those seen in clinical trials. Average increases of up to 13.9% in FEV1pp were reported in people receiving ETI [10, 11, 33] and up to 6.8% in people receiving TI [34, 35], where such increases were often as significant regardless of previous modulator treatment [11, 33]. The modest increases in lung function seen in our study are likely attributed to participants having more severe lung disease at baseline when compared with patients in pivotal clinicals. Additionally, the decision to select the “best” FEV1pp in the 12 months prior to commencement for comparison with postcommencement values may not recognize deterioration within the year before CFTR modulator commencement; therefore, less drastic increases in lung function may have been observed. Nevertheless, the large reduction in exacerbation frequency and sweat chloride and increase in BMI posttreatment were comparable to literature [10, 33] and suggest significant clinical improvement in this cohort after CFTR modulator commencement.

Investigations evaluating changes in CF lung microbiology after ETI treatment are limited to a number of relatively small studies that often lack consistency due to cohort demographics. These studies have reported increases in lung microbiota alpha diversity and decreases in *P. aeruginosa* relative abundance and load to varying levels that imply a shift in bacterial ecology [17, 33, 36, 37]. However, all studies consistently show that *P. aeruginosa* is not eradicated in all or a large proportion of patients [33, 36], as corroborated in observational studies reporting that 54% to 77% of pwCF remain *P. aeruginosa* culture positive up to 1 year of ETI modulation [14, 38, 39].

In this study, we have further demonstrated that not only do pwCF remain colonized with *P. aeruginosa* after CFTR modulation but the same clonal lineages persist, as opposed to the eradication of preexisting strains and establishment of new infections [7]. From our knowledge, only 1 other investigation has reported the persistence of *P. aeruginosa* lineages after 2 years of treatment with the first available modulator ivacaftor for treatment of G551D mutations [16]. Despite those authors performing a more comprehensive analysis of MLST frequencies at a population level, our findings of sequence type persistence are consistent with their study.

A likely explanation for *P. aeruginosa* persistence is irreversible structural damage that continues to cause defective pathogen clearance, especially in our cohort of pwCF with advanced lung disease. A study of 13 pwCF with severe lung disease reported reductions in pulmonary damage and bronchial destruction, yet signs of structural damage in the airways, such as scarring lesions and bronchiectasis, remained 1 year after treatment with ETI [38]. Individuals with other structural lung conditions, including non-CF bronchiectasis and chronic obstructive pulmonary disease, are commonly colonized by *P. aeruginosa* [40, 41]. Additionally, there have been small incidences where *P. aeruginosa* is acquired after CFTR modulator commencement, suggesting that lung damage remains a risk factor for infection [15, 17].

*P. aeruginosa* is well known for its hypermutable nature and ability to adapt to the harsh CF lung environment [30]. While structural damage is often sustained [38], multiple studies have reported important changes in the CF lung after modulation, such as changes in sputum load, viscosity, metabolomic composition, and pH [17, 36]. We found that *P. aeruginosa* accumulated mutations more frequently in previously described pathoadaptive genes but less often in antibiotic-resistant genes after CFTR modulation. This may indicate continual mutation of common chronic genotypes in these heterogenetic *P. aeruginosa* populations, as well as retention and lack of accumulation of additional antibiotic-resistant mechanisms due to reduced antibiotic exposure. The identification of genes mutated across a large proportion of lineages may indicate selection of adaptive mutants. Key genes were identified that play important roles in bacterial aggregation, alginate production, and biofilm formation: *cdrA*, *ppkA*, *mucA*, *pslA*, *pelA*, and *algG* [31, 42]. Genes encoding secreted effectors, including *tle5b* and *lepA*, were identified, as well as *pvdA*, *pvdL*, and *pchE*, which contribute to pyoverdine synthesis and iron acquisition [31]. Mutation of these genes may allow for continued survival and competition in the modulated CF lung. Despite the potential impact of the mutations reported, chronic *P. aeruginosa* phenotypes were sustained after CFTR modulation, including mucoid morphology for promoting biofilm formation, lack of O-antigen expression for immune evasion, and antibiotic resistance. Therefore, these *P. aeruginosa* lineages are not phenotypically changing or reverting back to more virulent acute phenotypes in this changed lung environment within this study time frame.

Limitations to this study include the small cohort size and the inconsistency of time points and duration, which were attributed to the coronavirus pandemic, where participants did not come to outpatient clinics consistently and research sampling could not always be undertaken. Only spontaneous sputum samples were collected from pwCF receiving compassionate access ETI, which resulted in this cohort including individuals with more advanced lung disease. Future studies with induced sputum sampling will allow for pathogen surveillance on a larger proportion of pwCF with varying disease severities, allowing conclusions to be drawn on *P. aeruginosa* persistence after CFTR modulation from a more representative cohort. Larger multicenter as well as international studies will further determine whether our findings are exclusive to common endemic strains found in Australia or relevant to all *P. aeruginosa* infections in pwCF after CFTR modulation.

One scientist performed bacterial isolations from CF sputum to limit variability in *P. aeruginosa* isolation, yet *P. aeruginosa* populations may not have been completely captured by taking only morphologically distinct colonies per time point. A recent study showed that 75 *P. aeruginosa* colonies from the same CF sputum sample can have between 31 and 4592 unique single-nucleotide polymorphisms within a population, highlighting the genomic diversity of *P. aeruginosa* and limitations in single-isolate sampling [43]. Therefore, collection of more *P. aeruginosa* colonies per time point are required to make more robust conclusions on *P. aeruginosa* adaptation and to investigate the impact of these mutations further. Despite this, we were still able to show that dominating clonal lineages with the same chronic phenotypes were persisting after CFTR modulation.

Despite the small sample size, the findings in this study have given important insights into the nature of chronic *P. aeruginosa* infection in pwCF with advanced lung disease after CFTR modulation. Persistence of the same chronic clonal lineages may indicate that initial improvements in health and systemic inflammation may slow lung disease progression; however, existing structural damage will allow for infection persistence and continued lung injury. Therefore, future research and clinical care should remain focused on *P. aeruginosa* as well as other lung infections in this post–modulator era. CFTR modulators will be instrumental for younger pwCF to prevent pathogen colonization and subsequent progressive lung disease.

## Supplementary Data

Supplementary materials are available at *The Journal of Infectious Diseases* online (http://jid.oxfordjournals.org/). Supplementary materials consist of data provided by the author that are published to benefit the reader. The posted materials are not copyedited. The contents of all supplementary data are the sole responsibility of the authors. Questions or messages regarding errors should be addressed to the author.
