# Supplementary material for: Impact of CFTR Modulation on Pseudomonas aeruginosa Infection in People With Cystic Fibrosis
Source: J Infect Dis. 2024 Mar 5;230(3):e536–47. doi: 10.1093/infdis/jiae051 (PMC11420785; doi:10.1093/infdis/jiae051)
Supplement: jiae051_Supplementary_Data [file jiae051_supplementary_data.zip › Supplementary_data_1 final.docx]

**Supplementary for:**

**Impact of CFTR modulation on *Pseudomonas aeruginosa* infection in people with cystic fibrosis**

Emma L. Ledger^1^, Daniel J. Smith^2,3^, Jing Jie Teh^1^, Michelle E. Wood^3^, Page E. Whibley^3^, Mark Morrison^1,4^, Joanna B. Goldberg^5^, David W. Reid^2,4,6^, Timothy J. Wells^#1,4^

^1^Frazer Institute, Faculty of Medicine, the University of Queensland, Brisbane, Australia

^2^Northside Clinical Unit, the University of Queensland, Brisbane, Australia

^3^Adult Cystic Fibrosis Centre, the Prince Charles Hospital, Brisbane, Australia

^4^Australian Infectious Diseases Research Centre, Brisbane, Australia

^5^Emory University, Atlanta, United States of America

^6^QIMR Berghofer Medical Research Institute, Brisbane, Australia

**Supplementary methods**

Whole genome sequencing continued

Microbes NG provided in-house bioinformatic services including read trimming with Trimmomatic[1], de novo assembly using SPAdes[2], genome annotation with Prokka[3] and quality assessment at a read and assembled genome level with BWA-MEM[4] (Table S1). Once sequencing data was received, genome assembly quality was also assessed using CheckM (v1.1.3)[5]. All genomes had a completeness of ≥95% and contamination score of ≤5% and were considered high quality and included in downstream genomics[5] (Table S1).

Comparative genomics continued

Approximately-maximum-likelihood phylogenetic trees were generated using FastTree software (<http://www.microbesonline.org/fasttree/>) and figures created in iTOL[20]. Tree is rooted to *P. aeruginosa* PAO1 reference strain (assembly accession: GCF_000006765.1[21]). Average nucleotide identity matrices (ANI) were generated on the EDGAR software platform using a BLASTN comparison of the genome sequences [21].

Variant calling

We utilised Snippy 4.6.0 (<https://github.com/tseemann/snippy>) to call variants including SNPs and indel mutations from the whole genome shotgun sequences of 90 isolates from persisting clonal lineages (14 clonal lineages from 11 subjects) against the PAO1 reference genome[6]. Mutations were filtered to exclude core mutations that differentiated each lineage from PAO1 and identify mutations that were unique to strains isolated after CFTR modulation (supplementary data 3 document for annotated filtered mutations). For this analysis mucoid strains isolated post modulation were excluded if no mucoid isolate was collected at baseline to avoid identifying mucoid mutations that were solely due to isolation limitations. Genes impacted by at least one unique non-synonymous (missense or nonsense), modifier indel (insertion or deletion) and/or frameshift mutation were identified in strains isolated after CFTR modulation. We performed a mutation enrichment of identified genes adapted from Eklöf et al[7] using a Fisher’s exact text of a collection of pathoadaptive genes (n = 159)[8-13] and antibiotic resistance genes (n = 185)[14-17] previously described (Table S5,6). To investigate potential positive selection of mutations, affected genes were compared between lineages and participants, and the PseudoCAP functional category[18] were assigned to genes mutated in over 42% (≥6/14, 37 identified genes) of clonal lineages (Table S4). Data was presented using pheatmap package in R. Unique mutations in *mutS, mutL* and *uvrD* were identified and non-synonymous mutations enumerated within all isolates from all lineages to determine potential hypermutator strains[19-22] (Table S7, supplementary data 4 for all annotated mutations). The number of mutations in these mutator-associated genes were compared between lineages that clustered together in number of genes commonly mutated and had a large proportion of overall mutations accumulated after CFTR modulation (>400, n = 6) to lineages with significantly less (<400) (Fig. S4).

O-antigen expression

Lipopolysaccharide (LPS) was first extracted from all 105 *P. aeruginosa* isolates and O-antigen typed control strains. In brief, overnight LB cultures were pelleted and resuspended in lysis buffer (0.05 M Tris-HCl, 30 % sodium dodecyl sulfate (SDS), 1 M 2-mercaptoethanol). The lysed suspension was boiled for 5 mins, frozen at -80°C, heated again for 5 mins and centrifuged at 20,000 × g for 5 mins. Proteinase K (Invitrogen^TM^) was added to the supernatant to a final concentration of 250 µg/mL and incubated in a 60 °C water bath for 1 hr. Extracts were boiled for 10 mins and stored at -20°C until use.

O-antigen expression was determined through SDS-polyacrylamide gel electrophoresis (PAGE) and western blotting with polyvalent *P. aeruginosa* O-antigen specific anti-serum (Denka Company Limited, 200372). LPS extracts were diluted in 4 × NuPAGE™ LDS sample loading buffer (Invitrogen™) and run on NuPAGE™ 4 – 12 % Bis-Tris pre-cast gels (Invitrogen™) with SeeBlue Plus2 pre-stained ladder (Invitrogen™) and control strains at ~100 V for 60 mins. Gels were transferred to polyvinylidene difluoride membranes via the iBlot 2 Dry blotting system (Invitrogen™) as per manufacturer’s instructions (20 V for 1 min, 23 V for 4 min, 25 V for 2 min). Membranes were blocked in blotto (5 % skim milk powder, 1 × tris-buffered saline (TBS)) then incubated in pooled polyvalent antisera specific for all IATS *P. aeruginosa* O-antigen groups (Denka Company Limited, 213556, 213563, 213570, each diluted 1:10,000 in blotto). This was followed by incubation with anti-rabbit conjugated – alkaline phosphatase (1:10,000, Sigma-Aldrich #A3687). All incubations were at room temperature for 1 hr, rocking. In between incubations the membranes were washed three times with TBST (1 × TBS, 0.1 % Tween20) wash buffer for 5 mins, rocking and developed with nitro-blue tetrazolium and 5-bromo-4-chloro-3’- indolyphosphate for 20 minutes (ThermoScientific).

Antibiotic susceptibility testing continued

To determine antibiotic susceptibility profiles of *P. aeruginosa* isolates, disc diffusion was performed as per the Clinical Laboratory Standards Institute (CLSI) guidelines and standards [23]. Isolates and control strain ATCC 27853 were grown overnight in Mueller-Hinton broth at 37 °C and shaking at 220 rpm. Cultures were diluted to 0.5 MacFarland standard and a lawn streaked onto Mueller-Hinton agar using sterile swabs. The following antibiotic disks (Oxoid^TM^) were stamped onto the agar plates including Amikacin (AK30), Ciproflaxin (CIP5), Tobramycin (TOB10), Ceftazidime (CAZ30), Aztreonam (ATM30), Meropenem (MEM10), Cefepime (FEP30), Piperacillin-tazobactam (TZP110) and colistin (CT10) and allowed to incubate for 18hrs at 35 °C (up to 48hrs was required for some slow growing mucoid isolates). Zones of clearance were measured, and diameters compared to CLSI breakpoints to classify isolates as sensitive, intermediate, or resistant. Diameters of quality control strain ATCC 27853 were within acceptable windows for all experiments.

**
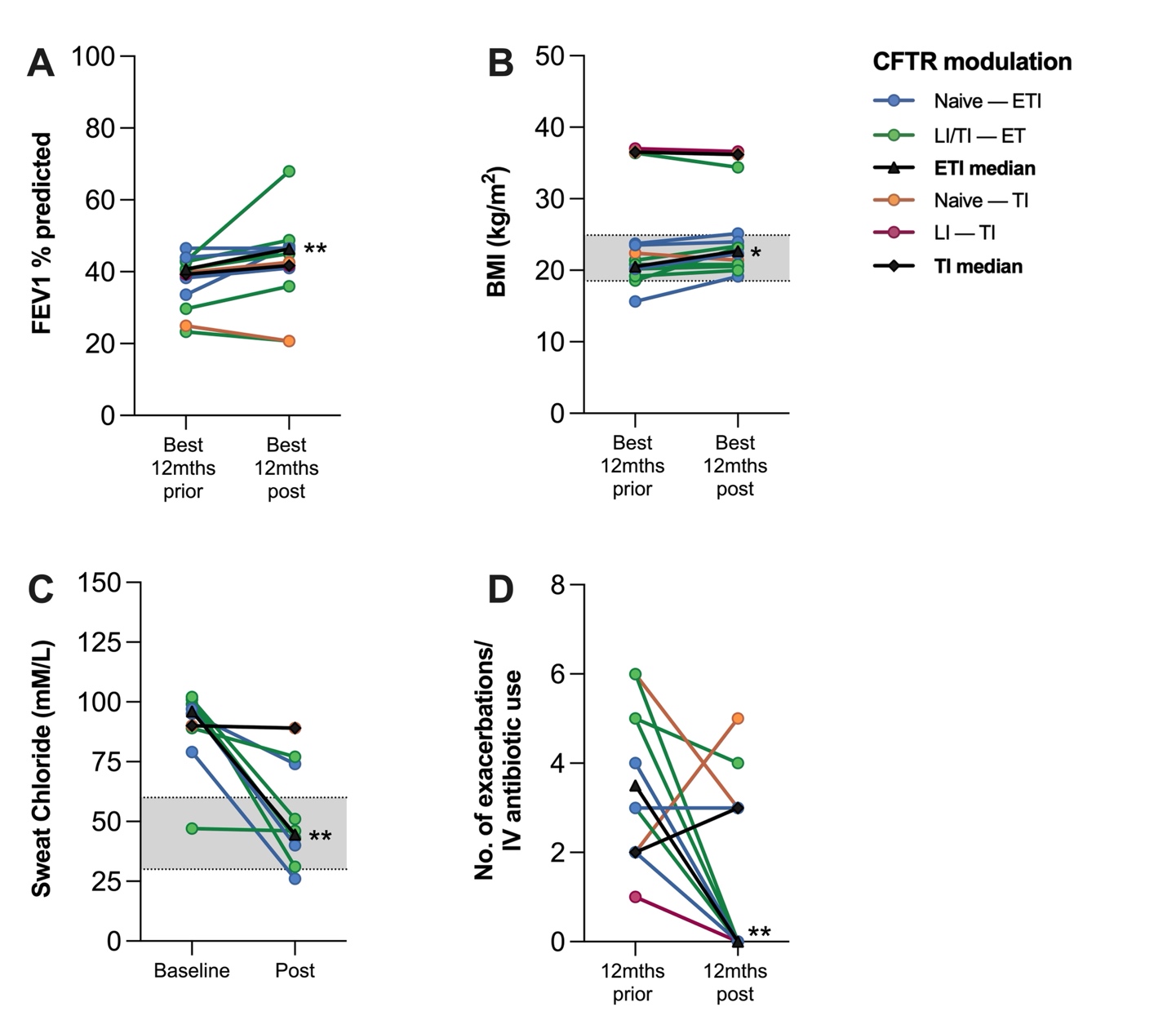
**

**Supplementary Figure 1. Individual changes in clinical parameters in the 12 months prior and post CFTR modulator therapy in people with cystic fibrosis.** Changes in A) Lung function (forced expiratory volume in one second (FEV1) percent predicted), B) Body mass index (BMI) C) Sweat Chloride and D) Exacerbation frequency/IV antibiotic use in the 12 months prior to 12 months post CFTR modulator therapy. Colours indicate CFTR modulator therapy characteristics. Black symbols represent the median of ETI (n = 10) and TI groups (n = 3). Two participants went from Naive to TI and transitioned to ETI and were also included in the LI/TI to ETI group and therefore represented twice on graphs (n = 13). Grey shaded regions represent the B) healthy BMI range (18.5 – 24.9) and C) the sweat chloride borderline values (31 – 60) where higher is diagnostic for CF and lower is considered a healthy individual. Significance between time points was determined with a paired Wilcoxon signed rank test of the ETI group only, * <0.05, ** <0.01 (FEV1 % predicted p = 0.0098, BMI p = 0.0391, sweat chloride p = 0.0078, exacerbations p = 0.0039).

**
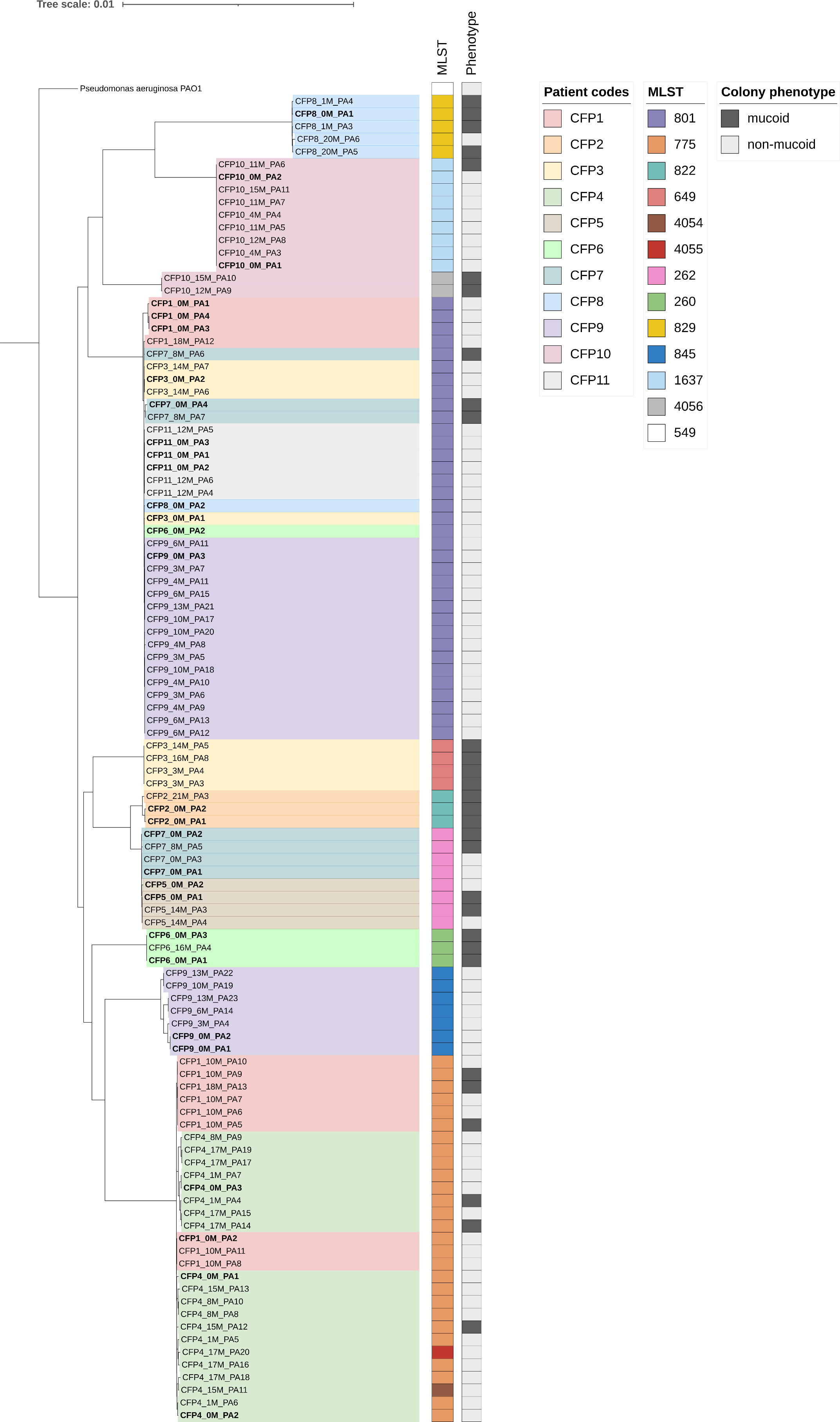
**

**Supplementary Figure 2. Phylogeny of longitudinal *P. aeruginosa* isolates.** Approximate-maximum-likelihood phylogenetic tree rooted to *P. aeruginosa* PAO1 reference strain. Shaded branches indicate isolates from each participant. Branch labels denote patient code _ months post CFTR modulator _ isolate number. Bolded labels highlight baseline isolates. Colour strips indicate strain multi-locus sequence type (MLST) and mucoid colony morphology phenotype.

**Supplementary Figure 3. Average nucleotide identity (ANI) between longitudinal *P. aeruginosa* isolates from each person with cystic fibrosis.** Heatmaps of the percentage ANI of all *P. aeruginosa* isolates.


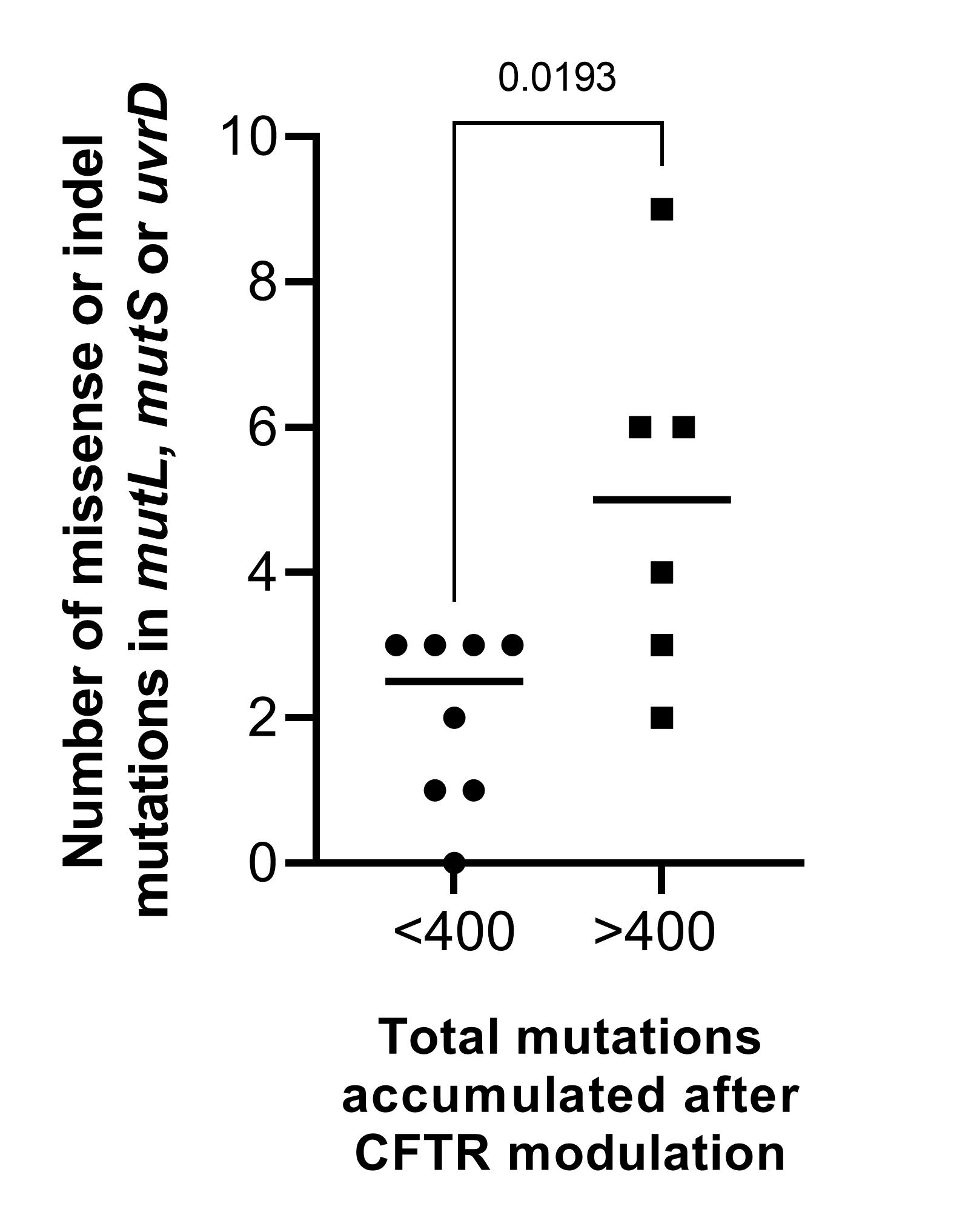


**Supplementary Figure 4. Lineages with over 400 overall mutations accumulated after CFTR modulation had more mutations in *mutS, mutL* and *uvrD* compared to lineages with less overall accumulated mutations.** Black line indicates the median. Significance between groups was determined via an unpaired Mann-Whitney t-test with the P-value displayed on the graph.

**Supplementary Table 1. Genome assembly statistics of *P. aeruginosa* isolates in this study**

| **NCBI BioSample** | **NCBI genome accession** | **NCBI strain name** | **Isolate code**  **(patient code_time point_isolate number)** | **Mean coverage** | **# contigs** | **Total length** | **GC (%)** | **N50 (bp)** | **CheckM** | |
| --- | --- | --- | --- | --- | --- | --- | --- | --- | --- | --- |
|  |  |  |  |  |  |  |  |  | **Completeness (%)** | **Contamination (%)** |
| SAMN37687486 | JAWDTL000000000 | CFP1_1 | **CFP11_0M_PA1** | 67.1917 | 42 | 6374008 | 66.39 | 321919 | 99.68 | 0.11 |
| SAMN37687487 | JAWDTK000000000 | CFP1_2 | **CFP11_0M_PA2** | 64.773 | 32 | 6377902 | 66.39 | 796911 | 99.68 | 0.15 |
| SAMN37687488 | JAWDTJ000000000 | CFP1_3 | **CFP11_0M_PA3** | 68.0872 | 31 | 6379874 | 66.39 | 796266 | 99.68 | 0.43 |
| SAMN37687489 | JAWDTI000000000 | CFP1_4 | **CFP9_6M_PA12** | 105.189 | 28 | 6380542 | 66.39 | 804536 | 99.68 | 0.11 |
| SAMN37687490 | JAWDTH000000000 | CFP1_5 | **CFP9_6M_PA13** | 85.2142 | 33 | 6381646 | 66.4 | 665188 | 99.68 | 0.11 |
| SAMN37687491 | JAWDTG000000000 | CFP1_6 | **CFP9_6M_PA14** | 101.664 | 15 | 6090732 | 66.32 | 670116 | 99.4 | 0.11 |
| SAMN37687492 | JAWDTF000000000 | CFP1_7 | **CFP9_6M_PA15** | 155.187 | 29 | 6377829 | 66.4 | 804542 | 99.68 | 0.11 |
| SAMN37687493 | JAWDTE000000000 | CFP1_8 | **CFP9_6M_PA16** | 117.487 | 27 | 6380880 | 66.4 | 804535 | 99.68 | 0.11 |
| SAMN37687494 | JAWDTD000000000 | CFP1_9 | **CFP1_10M_PA5** | 84.6317 | 36 | 6258861 | 66.56 | 425564 | 99.68 | 0.11 |
| SAMN37687495 | JAWDTC000000000 | CFP1_10 | **CFP1_10M_PA6** | 100.625 | 33 | 6260947 | 66.56 | 454361 | 99.68 | 0.11 |
| SAMN37687496 | JAWDTB000000000 | CFP1_11 | **CFP1_10M_PA7** | 101.401 | 34 | 6261123 | 66.56 | 615152 | 99.68 | 0.11 |
| SAMN37687497 | JAWDTA000000000 | CFP1_12 | **CFP1_10M_PA8** | 97.0073 | 47 | 6168405 | 66.48 | 567624 | 99.51 | 0.11 |
| SAMN37687498 | JAWDSZ000000000 | CFP1_13 | **CFP1_10M_PA9** | 91.5108 | 37 | 6259675 | 66.56 | 614757 | 99.68 | 0.11 |
| SAMN37687499 | JAWDSY000000000 | CFP2_1 | **CFP1_10M_PA10** | 112.667 | 82 | 6294687 | 66.48 | 615151 | 99.68 | 0.74 |
| SAMN37687500 | JAWDSX000000000 | CFP2_2 | **CFP1_10M_PA11** | 76.6289 | 19 | 6149979 | 66.55 | 738708 | 99.51 | 0.14 |
| SAMN37687501 | JAWDSW000000000 | CFP2_3 | **CFP10_12M_PA8** | 59.1744 | 27 | 6261751 | 66.59 | 756996 | 99.68 | 0.11 |
| SAMN37687502 | JAWDSV000000000 | CFP3_1 | **CFP10_12M_PA9** | 107.279 | 23 | 6203931 | 66.63 | 914424 | 99.49 | 0.11 |
| SAMN37687503 | JAWDSU000000000 | CFP3_2 | **CFP7_8M_PA5** | 73.3129 | 29 | 6057247 | 66.52 | 718049 | 99.35 | 0.11 |
| SAMN37687504 | JAWDST000000000 | CFP3_3 | **CFP7_8M_PA6** | 65.3209 | 26 | 6378891 | 66.4 | 804525 | 99.68 | 0.11 |
| SAMN37687505 | JAWDSS000000000 | CFP3_4 | **CFP7_8M_PA7** | 64.109 | 35 | 6317253 | 66.41 | 467222 | 99.68 | 0.11 |
| SAMN37687506 | JAWDSR000000000 | CFP3_5 | **CFP3_14M_PA5** | 128.898 | 30 | 6331165 | 66.53 | 770697 | 99.68 | 0.11 |
| SAMN37687507 | JAWDSQ000000000 | CFP3_6 | **CFP3_14M_PA6** | 88.9383 | 36 | 6389210 | 66.38 | 766960 | 99.68 | 0.11 |
| SAMN37687508 | JAWDSP000000000 | CFP3_7 | **CFP3_14M_PA7** | 115.709 | 26 | 6382997 | 66.4 | 1346622 | 99.68 | 0.11 |
| SAMN37687509 | JAWDSO000000000 | CFP3_8 | **CFP6_0M_PA1** | 98.3975 | 33 | 6342124 | 66.43 | 468415 | 99.68 | 0.11 |
| SAMN37687510 | JAWDSN000000000 | CFP4_1 | **CFP1_0M_PA1** | 76.3612 | 30 | 6346863 | 66.42 | 797631 | 99.19 | 0.11 |
| SAMN37687511 | JAWDSM000000000 | CFP4_2 | **CFP1_0M_PA2** | 109.666 | 23 | 6202337 | 66.58 | 770102 | 99.5 | 0.11 |
| SAMN37687512 | JAWDSL000000000 | CFP4_3 | **CFP1_0M_PA3** | 98.7889 | 31 | 6346893 | 66.42 | 769377 | 99.35 | 0.11 |
| SAMN37687513 | JAWDSK000000000 | CFP4_4 | **CFP1_0M_PA4** | 81.4139 | 28 | 6346031 | 66.42 | 769379 | 99.35 | 0.11 |
| SAMN37687514 | JAWDSJ000000000 | CFP4_5 | **CFP3_0M_PA1** | 87.6067 | 27 | 6377427 | 66.39 | 803946 | 99.68 | 0.11 |
| SAMN37687515 | JAWDSI000000000 | CFP4_6 | **CFP3_0M_PA2** | 105.052 | 26 | 6378891 | 66.4 | 804534 | 99.51 | 0.11 |
| SAMN37687516 | JAWDSH000000000 | CFP4_7 | **CFP6_0M_PA2** | 65.9513 | 25 | 6380132 | 66.4 | 785287 | 99.68 | 0.11 |
| SAMN37687517 | JAWDSG000000000 | CFP4_8 | **CFP10_15M_PA10** | 92.7125 | 29 | 6204954 | 66.63 | 410583 | 99.59 | 0.22 |
| SAMN37687518 | JAWDSF000000000 | CFP4_9 | **CFP10_15M_PA11** | 99.1088 | 20 | 6269427 | 66.6 | 756995 | 99.68 | 0.11 |
| SAMN37687519 | JAWDSE000000000 | CFP4_10 | **CFP9_0M_PA1** | 108.467 | 15 | 6151284 | 66.34 | 910344 | 99.19 | 0.13 |
| SAMN37687520 | JAWDSD000000000 | CFP4_11 | **CFP6_0M_PA3** | 136.973 | 30 | 6388089 | 66.38 | 565338 | 99.68 | 0.27 |
| SAMN37687521 | JAWDSC000000000 | CFP4_12 | **CFP9_0M_PA2** | 131.145 | 12 | 6150709 | 66.34 | 910340 | 99.19 | 0.11 |
| SAMN37687522 | JAWDSB000000000 | CFP4_13 | **CFP9_0M_PA3** | 87.1575 | 32 | 6373142 | 66.39 | 780979 | 99.68 | 0.11 |
| SAMN37687523 | JAWDSA000000000 | CFP4_14 | **CFP9_3M_PA4** | 82.0981 | 18 | 6151008 | 66.33 | 910436 | 99.19 | 0.11 |
| SAMN37687524 | JAWDRZ000000000 | CFP4_15 | **CFP9_3M_PA5** | 71.5354 | 32 | 6378513 | 66.39 | 804239 | 99.68 | 0.23 |
| SAMN37687525 | JAWDRY000000000 | CFP4_16 | **CFP9_3M_PA6** | 99.0015 | 33 | 6379412 | 66.39 | 780978 | 99.68 | 0.11 |
| SAMN37687526 | JAWDRX000000000 | CFP4_17 | **CFP9_3M_PA7** | 95.7285 | 27 | 6381062 | 66.4 | 804542 | 99.68 | 0.11 |
| SAMN37687527 | JAWDRW000000000 | CFP4_18 | **CFP9_4M_PA8** | 123.354 | 32 | 6377705 | 66.4 | 804530 | 99.68 | 0.11 |
| SAMN37687528 | JAWDRV000000000 | CFP4_19 | **CFP9_4M_PA9** | 99.2227 | 29 | 6381213 | 66.4 | 804535 | 99.68 | 0.11 |
| SAMN37687529 | JAWDRU000000000 | CFP4_20 | **CFP9_4M_PA10** | 55.9501 | 40 | 6379374 | 66.39 | 780876 | 99.68 | 0.82 |
| SAMN37687530 | JAWDRT000000000 | CFP5_1 | **CFP9_4M_PA11** | 31.4616 | 43 | 6373946 | 66.39 | 467694 | 99.68 | 0.11 |
| SAMN37687531 | JAWDRS000000000 | CFP5_2 | **CFP9_10M_PA17** | 150.038 | 28 | 6377814 | 66.4 | 804542 | 99.68 | 0.11 |
| SAMN37687532 | JAWDRR000000000 | CFP5_3 | **CFP9_10M_PA18** | 94.8293 | 31 | 6383205 | 66.4 | 804239 | 99.68 | 0.11 |
| SAMN37687533 | JAWDRQ000000000 | CFP5_4 | **CFP9_10M_PA19** | 98.4613 | 28 | 6283892 | 66.38 | 667454 | 99.51 | 0.11 |
| SAMN37687534 | JAWDRP000000000 | CFP6_1 | **CFP9_10M_PA20** | 99.6195 | 33 | 6379065 | 66.39 | 804534 | 99.68 | 0.11 |
| SAMN37687535 | JAWDRO000000000 | CFP6_2 | **CFP4_0M_PA1** | 111.747 | 34 | 6229686 | 66.55 | 454299 | 99.05 | 0.11 |
| SAMN37687536 | JAWDRN000000000 | CFP6_3 | **CFP4_0M_PA2** | 97.5446 | 22 | 6198995 | 66.57 | 567519 | 99.51 | 0.11 |
| SAMN37687537 | JAWDRM000000000 | CFP6_4 | **CFP4_0M_PA3** | 94.406 | 25 | 6156338 | 66.55 | 508028 | 98.94 | 0.43 |
| SAMN37687538 | JAWDRL000000000 | CFP7_1 | **CFP4_8M_PA8** | 110.383 | 28 | 6225689 | 66.55 | 664123 | 99.4 | 0.11 |
| SAMN37687539 | JAWDRK000000000 | CFP7_2 | **CFP4_8M_PA9** | 75.1718 | 35 | 6150024 | 66.55 | 657440 | 98.86 | 0.11 |
| SAMN37687540 | JAWDRJ000000000 | CFP7_3 | **CFP4_8M_PA10** | 89.823 | 33 | 6225926 | 66.55 | 664123 | 99.4 | 0.11 |
| SAMN37687541 | JAWDRI000000000 | CFP7_4 | **CFP10_0M_PA1** | 117.588 | 18 | 6267930 | 66.6 | 766669 | 99.68 | 0.11 |
| SAMN37687542 | JAWDRH000000000 | CFP7_5 | **CFP10_0M_PA2** | 95.8317 | 21 | 6263821 | 66.6 | 766976 | 99.68 | 0.11 |
| SAMN37687543 | JAWDRG000000000 | CFP7_6 | **CFP5_14M_PA3** | 71.0323 | 25 | 6058260 | 66.52 | 780397 | 99.35 | 0.11 |
| SAMN37687544 | JAWDRF000000000 | CFP7_7 | **CFP5_14M_PA4** | 101.3 | 20 | 6058978 | 66.52 | 904904 | 99.35 | 0.11 |
| SAMN37687545 | JAWDRE000000000 | CFP8_1 | **CFP5_0M_PA1** | 92.286 | 23 | 6062911 | 66.51 | 936862 | 99.35 | 0.11 |
| SAMN37687546 | JAWDRD000000000 | CFP8_2 | **CFP5_0M_PA2** | 93.2291 | 21 | 6058190 | 66.51 | 720432 | 99.35 | 0.11 |
| SAMN37687547 | JAWDRC000000000 | CFP8_3 | **CFP9_13M_PA21** | 65.7506 | 32 | 6372627 | 66.4 | 780977 | 99.68 | 0.11 |
| SAMN37687548 | JAWDRB000000000 | CFP8_4 | **CFP9_13M_PA22** | 115.526 | 26 | 6284531 | 66.38 | 667454 | 99.51 | 0.11 |
| SAMN37687549 | JAWDRA000000000 | CFP8_5 | **CFP9_13M_PA23** | 79.6212 | 17 | 6095179 | 66.32 | 670113 | 99.4 | 0.11 |
| SAMN37687550 | JAWDQZ000000000 | CFP8_6 | **CFP10_11M_PA5** | 104.437 | 19 | 6269040 | 66.6 | 766669 | 99.68 | 0.11 |
| SAMN37687551 | JAWDQY000000000 | CFP9_1 | **CFP10_11M_PA6** | 110.813 | 22 | 6266601 | 66.59 | 756995 | 99.68 | 0.11 |
| SAMN37687552 | JAWDQX000000000 | CFP9_2 | **CFP10_11M_PA7** | 102.153 | 17 | 6270340 | 66.6 | 766656 | 99.68 | 0.11 |
| SAMN37687553 | JAWDQW000000000 | CFP9_3 | **CFP3_16M_PA8** | 107.635 | 30 | 6332542 | 66.53 | 770913 | 99.68 | 0.11 |
| SAMN37687554 | JAWDQV000000000 | CFP9_4 | **CFP11_12M_PA4** | 43.7386 | 32 | 6374184 | 66.39 | 780788 | 99.68 | 0.11 |
| SAMN37687555 | JAWDQU000000000 | CFP9_5 | **CFP11_12M_PA5** | 46.6033 | 36 | 6378084 | 66.39 | 796266 | 99.68 | 0.11 |
| SAMN37687556 | JAWDQT000000000 | CFP9_6 | **CFP11_12M_PA6** | 61.9581 | 26 | 6377121 | 66.39 | 804535 | 99.68 | 0.11 |
| SAMN37687557 | JAWDQS000000000 | CFP9_7 | **CFP4_15M_PA11** | 101.092 | 25 | 6236405 | 66.55 | 554608 | 99.51 | 0.11 |
| SAMN37687558 | JAWDQR000000000 | CFP9_8 | **CFP4_15M_PA12** | 79.9593 | 28 | 6202722 | 66.57 | 668006 | 99.4 | 0.11 |
| SAMN37687559 | JAWDQQ000000000 | CFP9_9 | **CFP4_15M_PA13** | 82.6562 | 31 | 6135104 | 66.57 | 665995 | 99.51 | 0.11 |
| SAMN37687560 | JAWDQP000000000 | CFP9_10 | **CFP4_17M_PA14** | 91.9049 | 27 | 6152631 | 66.55 | 662563 | 99.02 | 0.11 |
| SAMN37687561 | JAWDQO000000000 | CFP9_11 | **CFP4_17M_PA15** | 114.342 | 24 | 6153523 | 66.55 | 662145 | 99.02 | 0.11 |
| SAMN37687562 | JAWDQN000000000 | CFP9_12 | **CFP4_17M_PA16** | 102.427 | 30 | 6230640 | 66.55 | 689077 | 99.4 | 0.26 |
| SAMN37687563 | JAWDQM000000000 | CFP9_13 | **CFP4_17M_PA17** | 112.324 | 25 | 6151982 | 66.55 | 727863 | 98.86 | 0.11 |
| SAMN37687564 | JAWDQL000000000 | CFP9_14 | **CFP4_17M_PA18** | 81.4598 | 31 | 6022344 | 66.54 | 664395 | 99.4 | 0.11 |
| SAMN37687565 | JAWDQK000000000 | CFP9_15 | **CFP4_17M_PA19** | 86.0654 | 35 | 6149095 | 66.55 | 454479 | 98.86 | 0.11 |
| SAMN37687566 | JAWDQJ000000000 | CFP9_16 | **CFP4_17M_PA20** | 89.8739 | 22 | 6240646 | 66.55 | 702749 | 99.24 | 0.11 |
| SAMN37687567 | JAWDQI000000000 | CFP9_17 | **CFP6_16M_PA4** | 113.322 | 32 | 6387857 | 66.38 | 666197 | 99.68 | 0.27 |
| SAMN37687568 | JAWDQH000000000 | CFP9_18 | **CFP1_18M_PA12** | 101.644 | 30 | 6269987 | 66.36 | 665176 | 99.68 | 0.11 |
| SAMN37687569 | JAWDQG000000000 | CFP9_19 | **CFP1_18M_PA13** | 107.327 | 31 | 6263009 | 66.56 | 508529 | 99.68 | 0.22 |
| SAMN37687570 | JAWDQF000000000 | CFP9_20 | **CFP2_21M_PA3** | 87.4602 | 27 | 6338867 | 66.49 | 498518 | 99.51 | 0.11 |
| SAMN37687571 | JAWDQE000000000 | CFP9_21 | **CFP2_0M_PA1** | 83.7827 | 22 | 6333328 | 66.51 | 765852 | 99.51 | 0.11 |
| SAMN37687572 | JAWDQD000000000 | CFP9_22 | **CFP2_0M_PA2** | 117.202 | 27 | 6334691 | 66.51 | 801282 | 99.19 | 0.11 |
| SAMN37687573 | JAWDQC000000000 | CFP9_23 | **CFP8_20M_PA5** | 66.6993 | 127 | 6735889 | 66.02 | 105893 | 99.35 | 0.43 |
| SAMN37687574 | JAWDQB000000000 | CFP10_1 | **CFP8_20M_PA6** | 79.0247 | 134 | 6735711 | 66.02 | 109715 | 99.35 | 0.43 |
| SAMN37687575 | JAWDQA000000000 | CFP10_2 | **CFP8_0M_PA1** | 84.8575 | 118 | 6730591 | 66.04 | 123248 | 99.35 | 0.43 |
| SAMN37687576 | JAWDPZ000000000 | CFP10_3 | **CFP8_0M_PA2** | 126.728 | 18 | 6138887 | 66.34 | 1346640 | 99.68 | 0.11 |
| SAMN37687577 | JAWDPY000000000 | CFP10_4 | **CFP8_1M_PA3** | 69.6973 | 131 | 6722311 | 66.05 | 116794 | 99.24 | 0.43 |
| SAMN37687578 | JAWDPX000000000 | CFP10_5 | **CFP8_1M_PA4** | 102.513 | 122 | 6738739 | 66.03 | 110161 | 99.35 | 0.43 |
| SAMN37687579 | JAWDPW000000000 | CFP10_6 | **CFP4_1M_PA4** | 114.33 | 22 | 6154867 | 66.55 | 926881 | 99.02 | 0.11 |
| SAMN37687580 | JAWDPV000000000 | CFP10_7 | **CFP4_1M_PA5** | 211.07 | 26 | 6237255 | 66.55 | 693848 | 99.51 | 0.11 |
| SAMN37687581 | JAWDPU000000000 | CFP10_8 | **CFP4_1M_PA6** | 98.0502 | 18 | 6200495 | 66.57 | 566151 | 99.51 | 0.11 |
| SAMN37687582 | JAWDPT000000000 | CFP10_9 | **CFP4_1M_PA7** | 118.091 | 19 | 6154810 | 66.55 | 667720 | 98.94 | 0.11 |
| SAMN37687583 | JAWDPS000000000 | CFP10_10 | **CFP3_3M_PA3** | 113.435 | 31 | 6332469 | 66.53 | 770913 | 99.68 | 0.27 |
| SAMN37687584 | JAWDPR000000000 | CFP10_11 | **CFP7_0M_PA1** | 76.0552 | 26 | 6059887 | 66.52 | 742178 | 99.35 | 0.11 |
| SAMN37687585 | JAWDPQ000000000 | CFP11_1 | **CFP3_3M_PA4** | 97.2143 | 25 | 6333719 | 66.53 | 770913 | 98.68 | 0.11 |
| SAMN37687586 | JAWDPP000000000 | CFP11_2 | **CFP7_0M_PA2** | 106.186 | 91 | 6107015 | 66.47 | 780390 | 99.35 | 0.22 |
| SAMN37687587 | JAWDPO000000000 | CFP11_3 | **CFP7_0M_PA3** | 61.1912 | 24 | 6058456 | 66.52 | 904900 | 99.35 | 0.11 |
| SAMN37687588 | JAWDPN000000000 | CFP11_4 | **CFP7_0M_PA4** | 35.1841 | 39 | 6314152 | 66.42 | 665710 | 99.59 | 0.31 |
| SAMN37687589 | JAWDPM000000000 | CFP11_5 | **CFP10_4M_PA3** | 92.9486 | 20 | 6270085 | 66.6 | 766669 | 98.68 | 0.11 |
| SAMN37687590 | JAWDPL000000000 | CFP11_6 | **CFP10_4M_PA4** | 97.8275 | 18 | 6268425 | 66.6 | 766669 | 98.68 | 0.11 |

**Supplementary Table 2. *P. aeruginosa* isolate characteristics and phenotypes**

| **NCBI genome accession** | **NCBI strain name** | **Isolate code (patient code_time point_isolate number)** | **Phenotype** | **O-antigen Serotype** | **O-antigen expression** | **MLST** | **Antibiotic Succeptibility** | | | | | | | | |
| --- | --- | --- | --- | --- | --- | --- | --- | --- | --- | --- | --- | --- | --- | --- | --- |
|  |  |  |  |  |  |  | **AK** | **CIP** | **TOB** | **CAZ** | **AZM** | **MEM** | **CT** | **FEP** | **TZP** |
| JAWDTL000000000 | CFP1_1 | **CFP1_0M_PA1** | non-mucoid | O6 | **No** | 801 | R | I | I | R | R | R | S | R | R |
| JAWDTK000000000 | CFP1_2 | **CFP1_0M_PA2** | non-mucoid | O3 | **No** | 775 | R | I | R | R | R | R | S | R | R |
| JAWDTJ000000000 | CFP1_3 | **CFP1_0M_PA3** | non-mucoid | O6 | **No** | 801 | R | I | I | R | R | R | S | R | I |
| JAWDTI000000000 | CFP1_4 | **CFP1_0M_PA4** | non-mucoid | O6 | **No** | 801 | R | I | R | R | R | R | S | R | R |
| JAWDTH000000000 | CFP1_5 | **CFP1_10M_PA5** | mucoid | O3 | **No** | 775 | S | I | R | R | R | R | S | R | R |
| JAWDTG000000000 | CFP1_6 | **CFP1_10M_PA6** | non-mucoid | O3 | **No** | 775 | S | S | R | R | R | R | S | R | I |
| JAWDTF000000000 | CFP1_7 | **CFP1_10M_PA7** | non-mucoid | O3 | **No** | 775 | S | S | R | R | R | R | S | I | S |
| JAWDTE000000000 | CFP1_8 | **CFP1_10M_PA8** | non-mucoid | O3 | **No** | 775 | R | I | R | R | R | R | S | R | R |
| JAWDTD000000000 | CFP1_9 | **CFP1_10M_PA9** | mucoid | O3 | **No** | 775 | S | I | R | R | R | I | S | R | I |
| JAWDTC000000000 | CFP1_10 | **CFP1_10M_PA10** | non-mucoid | O3 | **No** | 775 | S | I | R | R | R | R | S | S | I |
| JAWDTB000000000 | CFP1_11 | **CFP1_10M_PA11** | non-mucoid | O3 | **No** | 775 | I | S | S | R | R | R | S | R | R |
| JAWDTA000000000 | CFP1_12 | **CFP1_18M_PA12** | non-mucoid | O6 | **Yes** | 801 | R | R | R | R | R | R | S | R | R |
| JAWDSZ000000000 | CFP1_13 | **CFP1_18M_PA13** | mucoid | O3 | **No** | 775 | S | I | R | R | R | R | S | R | R |
| JAWDSY000000000 | CFP2_1 | **CFP2_0M_PA1** | mucoid | O6 | **Yes** | 822 | I | S | R | R | I | I | S | R | R |
| JAWDSX000000000 | CFP2_2 | **CFP2_0M_PA2** | mucoid | O6 | **Yes** | 822 | I | R | R | R | R | R | S | R | R |
| JAWDSW000000000 | CFP2_3 | **CFP2_21M_PA3** | mucoid | O6 | **Yes** | 822 | I | R | R | R | R | R | S | R | I |
| JAWDSV000000000 | CFP3_1 | **CFP3_0M_PA1** | non-mucoid | O6 | **No** | 801 | R | R | R | R | R | R | S | R | R |
| JAWDSU000000000 | CFP3_2 | **CFP3_0M_PA2** | non-mucoid | O6 | **No** | 801 | R | I | R | R | R | R | S | R | R |
| JAWDST000000000 | CFP3_3 | **CFP3_3M_PA3** | mucoid | O6 | **No** | 649 | R | R | S | S | S | S | S | S | S |
| JAWDSS000000000 | CFP3_4 | **CFP3_3M_PA4** | mucoid | O6 | **No** | 649 | R | R | S | S | S | S | S | S | S |
| JAWDSR000000000 | CFP3_5 | **CFP3_14M_PA5** | mucoid | O6 | **No** | 649 | R | R | S | S | S | S | S | S | S |
| JAWDSQ000000000 | CFP3_6 | **CFP3_14M_PA6** | non-mucoid | O6 | **No** | 801 | R | I | R | R | R | R | S | R | R |
| JAWDSP000000000 | CFP3_7 | **CFP3_14M_PA7** | non-mucoid | O6 | **No** | 801 | R | I | R | R | R | R | S | R | R |
| JAWDSO000000000 | CFP3_8 | **CFP3_16M_PA8** | mucoid | O6 | **No** | 649 | R | R | S | S | S | S | S | S | S |
| JAWDSN000000000 | CFP4_1 | **CFP4_0M_PA1** | non-mucoid | O3 | **No** | 775 | R | R | R | R | R | R | S | R | R |
| JAWDSM000000000 | CFP4_2 | **CFP4_0M_PA2** | non-mucoid | O3 | **No** | 775 | R | I | R | R | R | R | S | R | R |
| JAWDSL000000000 | CFP4_3 | **CFP4_0M_PA3** | non-mucoid | O3 | **No** | 775 | R | R | R | R | R | R | S | R | R |
| JAWDSK000000000 | CFP4_4 | **CFP4_1M_PA4** | mucoid | O3 | **Yes** | 775 | R | R | R | R | R | R | S | R | R |
| JAWDSJ000000000 | CFP4_5 | **CFP4_1M_PA5** | non-mucoid | O3 | **No** | 775 | R | R | R | R | R | R | S | R | R |
| JAWDSI000000000 | CFP4_6 | **CFP4_1M_PA6** | non-mucoid | O3 | **No** | 775 | R | I | R | R | R | R | S | R | I |
| JAWDSH000000000 | CFP4_7 | **CFP4_1M_PA7** | non-mucoid | O3 | **No** | 775 | R | R | R | R | R | R | S | R | R |
| JAWDSG000000000 | CFP4_8 | **CFP4_8M_PA8** | non-mucoid | O3 | **No** | 775 | R | I | I | R | R | R | S | R | R |
| JAWDSF000000000 | CFP4_9 | **CFP4_8M_PA9** | non-mucoid | O3 | **No** | 775 | I | R | I | R | R | R | S | R | R |
| JAWDSE000000000 | CFP4_10 | **CFP4_8M_PA10** | non-mucoid | O3 | **No** | 775 | R | I | R | R | R | R | S | R | R |
| JAWDSD000000000 | CFP4_11 | **CFP4_15M_PA11** | non-mucoid | O3 | **No** | 4054 | R | I | R | R | R | R | S | R | R |
| JAWDSC000000000 | CFP4_12 | **CFP4_15M_PA12** | mucoid | O3 | **No** | 775 | R | I | R | R | R | R | S | R | I |
| JAWDSB000000000 | CFP4_13 | **CFP4_15M_PA13** | non-mucoid | O3 | **No** | 775 | R | I | R | R | R | R | S | R | R |
| JAWDSA000000000 | CFP4_14 | **CFP4_17M_PA14** | mucoid | O3 | **No** | 775 | R | R | R | R | R | R | S | R | R |
| JAWDRZ000000000 | CFP4_15 | **CFP4_17M_PA15** | non-mucoid | O3 | **No** | 775 | R | R | I | R | R | R | S | R | S |
| JAWDRY000000000 | CFP4_16 | **CFP4_17M_PA16** | non-mucoid | O3 | **No** | 775 | R | R | R | R | R | R | S | R | R |
| JAWDRX000000000 | CFP4_17 | **CFP4_17M_PA17** | non-mucoid | O3 | **No** | 775 | R | R | R | R | R | R | S | R | R |
| JAWDRW000000000 | CFP4_18 | **CFP4_17M_PA18** | non-mucoid | O3 | **No** | 775 | R | I | R | R | R | R | S | R | R |
| JAWDRV000000000 | CFP4_19 | **CFP4_17M_PA19** | non-mucoid | O3 | **No** | 775 | R | R | R | R | R | R | S | R | R |
| JAWDRU000000000 | CFP4_20 | **CFP4_17M_PA20** | non-mucoid | O3 | **No** | 4055 | R | R | S | R | R | R | S | R | R |
| JAWDRT000000000 | CFP5_1 | **CFP5_0M_PA1** | mucoid | O6 | **No** | 262 | R | I | R | R | R | R | S | R | R |
| JAWDRS000000000 | CFP5_2 | **CFP5_0M_PA2** | non-mucoid | O6 | **No** | 262 | R | I | R | R | R | R | S | R | R |
| JAWDRR000000000 | CFP5_3 | **CFP5_14M_PA3** | mucoid | O6 | **No** | 262 | R | I | R | R | R | R | S | R | R |
| JAWDRQ000000000 | CFP5_4 | **CFP5_14M_PA4** | non-mucoid | O6 | **No** | 262 | R | I | R | R | R | R | S | R | R |
| JAWDRP000000000 | CFP6_1 | **CFP6_0M_PA1** | mucoid | O6 | **No** | 260 | R | R | I | I | S | S | S | S | S |
| JAWDRO000000000 | CFP6_2 | **CFP6_0M_PA2** | non-mucoid | O6 | **Yes** | 801 | R | I | I | R | R | R | S | R | I |
| JAWDRN000000000 | CFP6_3 | **CFP6_0M_PA3** | mucoid | O6 | **No** | 260 | R | R | I | S | S | S | S | S | S |
| JAWDRM000000000 | CFP6_4 | **CFP6_16M_PA4** | mucoid | O6 | **No** | 260 | R | R | S | S | S | S | S | S | S |
| JAWDRL000000000 | CFP7_1 | **CFP7_0M_PA1** | non-mucoid | O6 | **No** | 262 | R | R | R | R | R | R | S | R | R |
| JAWDRK000000000 | CFP7_2 | **CFP7_0M_PA2** | mucoid | O6 | **No** | 262 | R | R | I | R | R | R | S | R | R |
| JAWDRJ000000000 | CFP7_3 | **CFP7_0M_PA3** | non-mucoid | O6 | **No** | 262 | R | R | R | R | R | R | S | R | R |
| JAWDRI000000000 | CFP7_4 | **CFP7_0M_PA4** | mucoid | O6 | **No** | 801 | R | R | I | R | R | R | S | R | R |
| JAWDRH000000000 | CFP7_5 | **CFP7_8M_PA5** | mucoid | O6 | **No** | 262 | R | R | I | R | R | R | S | R | R |
| JAWDRG000000000 | CFP7_6 | **CFP7_8M_PA6** | mucoid | O6 | **No** | 801 | R | R | R | R | R | R | S | R | R |
| JAWDRF000000000 | CFP7_7 | **CFP7_8M_PA7** | mucoid | O6 | **No** | 801 | R | R | R | R | R | R | S | R | R |
| JAWDRE000000000 | CFP8_1 | **CFP8_0M_PA1** | mucoid | O6 | **No** | 829 | R | I | S | S | S | S | S | R | I |
| JAWDRD000000000 | CFP8_2 | **CFP8_0M_PA2** | non-mucoid | O6 | **No** | 801 | R | I | I | R | R | R | S | R | R |
| JAWDRC000000000 | CFP8_3 | **CFP8_1M_PA3** | mucoid | O6 | **No** | 829 | R | I | S | R | R | R | S | I | I |
| JAWDRB000000000 | CFP8_4 | **CFP8_1M_PA4** | mucoid | O6 | **No** | 829 | R | I | S | R | R | S | S | R | R |
| JAWDRA000000000 | CFP8_5 | **CFP8_20M_PA5** | mucoid | O6 | **No** | 829 | S | S | S | R | I | S | S | I | I |
| JAWDQZ000000000 | CFP8_6 | **CFP8_20M_PA6** | non-mucoid | O6 | **No** | 829 | R | S | S | R | R | S | S | R | I |
| JAWDQY000000000 | CFP9_1 | **CFP9_0M_PA1** | non-mucoid | O1 | **No** | 845 | I | R | S | R | R | R | S | R | R |
| JAWDQX000000000 | CFP9_2 | **CFP9_0M_PA2** | non-mucoid | O1 | **No** | 845 | I | R | S | R | R | R | S | R | R |
| JAWDQW000000000 | CFP9_3 | **CFP9_0M_PA3** | non-mucoid | O6 | **No** | 801 | R | R | R | R | R | R | S | R | R |
| JAWDQV000000000 | CFP9_4 | **CFP9_3M_PA4** | non-mucoid | O1 | **No** | 845 | R | I | I | R | R | R | S | R | R |
| JAWDQU000000000 | CFP9_5 | **CFP9_3M_PA5** | non-mucoid | O6 | **No** | 801 | R | R | S | R | R | R | S | R | I |
| JAWDQT000000000 | CFP9_6 | **CFP9_3M_PA6** | non-mucoid | O6 | **No** | 801 | R | R | R | R | R | R | S | R | R |
| JAWDQS000000000 | CFP9_7 | **CFP9_3M_PA7** | non-mucoid | O6 | **No** | 801 | R | R | R | R | R | R | S | R | R |
| JAWDQR000000000 | CFP9_8 | **CFP9_4M_PA8** | non-mucoid | O6 | **No** | 801 | R | R | R | R | R | R | S | R | R |
| JAWDQQ000000000 | CFP9_9 | **CFP9_4M_PA9** | non-mucoid | O6 | **No** | 801 | R | R | S | R | R | R | S | R | I |
| JAWDQP000000000 | CFP9_10 | **CFP9_4M_PA10** | non-mucoid | O6 | **No** | 801 | R | R | S | R | R | R | S | R | R |
| JAWDQO000000000 | CFP9_11 | **CFP9_4M_PA11** | non-mucoid | O6 | **No** | 801 | R | R | R | R | R | R | S | R | R |
| JAWDQN000000000 | CFP9_12 | **CFP9_6M_PA12** | non-mucoid | O6 | **No** | 801 | R | R | R | R | R | R | S | R | R |
| JAWDQM000000000 | CFP9_13 | **CFP9_6M_PA13** | non-mucoid | O6 | **No** | 801 | R | R | I | R | R | R | S | R | R |
| JAWDQL000000000 | CFP9_14 | **CFP9_6M_PA14** | non-mucoid | O1 | **No** | 845 | I | I | S | R | R | R | S | R | R |
| JAWDQK000000000 | CFP9_15 | **CFP9_6M_PA15** | non-mucoid | O6 | **No** | 801 | R | R | R | R | R | R | S | R | R |
| JAWDQJ000000000 | CFP9_16 | **CFP9_6M_PA16** | non-mucoid | O6 | **No** | 801 | R | R | I | R | R | R | S | R | R |
| JAWDQI000000000 | CFP9_17 | **CFP9_10M_PA17** | non-mucoid | O6 | **No** | 801 | R | R | R | R | R | R | S | R | R |
| JAWDQH000000000 | CFP9_18 | **CFP9_10M_PA18** | non-mucoid | O6 | **No** | 801 | R | R | S | R | R | R | S | R | R |
| JAWDQG000000000 | CFP9_19 | **CFP9_10M_PA19** | non-mucoid | O1 | **No** | 845 | R | R | S | R | R | R | S | R | R |
| JAWDQF000000000 | CFP9_20 | **CFP9_10M_PA20** | non-mucoid | O6 | **No** | 801 | R | R | I | R | R | R | S | R | R |
| JAWDQE000000000 | CFP9_21 | **CFP9_13M_PA21** | non-mucoid | O6 | **No** | 801 | R | R | R | R | R | R | S | R | R |
| JAWDQD000000000 | CFP9_22 | **CFP9_13M_PA22** | non-mucoid | O1 | **No** | 845 | S | R | S | R | R | R | S | R | R |
| JAWDQC000000000 | CFP9_23 | **CFP9_13M_PA23** | non-mucoid | O1 | **No** | 845 | R | R | S | R | R | R | S | R | R |
| JAWDQB000000000 | CFP10_1 | **CFP10_0M_PA1** | non-mucoid | O1 | **Yes** | 1637 | R | I | I | S | I | R | S | I | S |
| JAWDQA000000000 | CFP10_2 | **CFP10_0M_PA2** | non-mucoid | O1 | **Yes** | 1637 | R | S | S | R | R | S | S | I | R |
| JAWDPZ000000000 | CFP10_3 | **CFP10_4M_PA3** | non-mucoid | O1 | **Yes** | 1637 | R | I | S | S | I | R | S | S | I |
| JAWDPY000000000 | CFP10_4 | **CFP10_4M_PA4** | non-mucoid | O1 | **Yes** | 1637 | R | I | S | S | I | R | S | I | I |
| JAWDPX000000000 | CFP10_5 | **CFP10_11M_PA5** | non-mucoid | O1 | **Yes** | 1637 | R | R | S | S | I | R | S | I | S |
| JAWDPW000000000 | CFP10_6 | **CFP10_11M_PA6** | mucoid | O1 | **Yes** | 1637 | R | I | S | S | S | R | S | S | S |
| JAWDPV000000000 | CFP10_7 | **CFP10_11M_PA7** | non-mucoid | O1 | **Yes** | 1637 | R | I | S | S | I | R | S | S | S |
| JAWDPU000000000 | CFP10_8 | **CFP10_12M_PA8** | non-mucoid | O1 | **No** | 1637 | R | I | S | S | I | R | S | I | I |
| JAWDPT000000000 | CFP10_9 | **CFP10_12M_PA9** | mucoid | O6 | **No** | 4056 | R | R | R | R | R | R | S | R | R |
| JAWDPS000000000 | CFP10_10 | **CFP10_15M_PA10** | mucoid | O6 | **No** | 4056 | R | R | R | R | R | R | S | R | R |
| JAWDPR000000000 | CFP10_11 | **CFP10_15M_PA11** | non-mucoid | O1 | **Yes** | 1637 | I | S | S | R | R | S | S | I | R |
| JAWDPQ000000000 | CFP11_1 | **CFP11_0M_PA1** | non-mucoid | O6 | **No** | 801 | R | R | R | R | R | R | S | R | R |
| JAWDPP000000000 | CFP11_2 | **CFP11_0M_PA2** | non-mucoid | O6 | **No** | 801 | R | R | R | R | R | R | S | R | R |
| JAWDPO000000000 | CFP11_3 | **CFP11_0M_PA3** | non-mucoid | O6 | **No** | 801 | R | R | R | R | R | R | S | R | I |
| JAWDPN000000000 | CFP11_4 | **CFP11_12M_PA4** | non-mucoid | O6 | **No** | 801 | R | R | R | R | R | R | S | R | R |
| JAWDPM000000000 | CFP11_5 | **CFP11_12M_PA5** | non-mucoid | O6 | **No** | 801 | R | I | R | R | R | R | S | R | S |
| JAWDPL000000000 | CFP11_6 | **CFP11_12M_PA6** | non-mucoid | O6 | **No** | 801 | R | R | R | R | R | R | S | R | I |

Abbreviations: AK – Amikacin, CIP – Ciproflaxin, TOB – Tobramycin, CAZ – Ceftazidime, AZM – Aztreonam, MEM – Meropenem, CT – Colistin, FEP – Cefepime, TZP – Piperacillin-tazobactam

**Supplementary Table 3. Individual patient changes in clinical parameters from the 12months prior to the 12months post CFTR modulator therapy in people with cystic fibrosis**

| **CFTR modulator** | | **Patient code** | **Sex** | **Genotype** | | **Age** | **Best FEV1pp in 12mths** *(%)* | | | **Best BMI in 12mths** *(kg/m2)* | | | **Sweat Chloride** *(mM/L)* | | | **No. exacerbations in 12mths** | | |
| --- | --- | --- | --- | --- | --- | --- | --- | --- | --- | --- | --- | --- | --- | --- | --- | --- | --- | --- |
| Before study | This study |  |  | Allele 1 | Allele 2 |  | Prior | Post | Change | Prior | Post | Change | Baseline | Post | Change | Prior | Post | Change |
| None | ETI | CFP 1 | M | F508del | R347P | 30.6 | 38.3 | 41 | 2.8 | 15.7 | 19.1 | 3.5 | 79 | 26 | -53 | 2 | 0 | -2 |
| None | ETI | CFP 2 | M | F508del | R1162X | 30.1 | 33.6 | 47.1 | 13.5 | 23.7 | 25.2 | 1.4 | 95 | 74 | -21 | 4 | 0 | -4 |
| None | ETI | CFP 3 | F | F508del | G542X | 27.1 | 44 | 46.2 | 2.2 | 20.2 | 22.3 | 2.1 | 97 | 40 | -57 | 3 | 3 | 0 |
| None | ETI | CFP 4 | F | F508del | 2185delA | 45.3 | 46.5 | 46.5 | 0 | 23.5 | 24 | 0.5 | N.D | N.D | N.D | 2 | 0 | -2 |
| LI | ETI | CFP 5 | M | F508del | F508del | 28.4 | 43.1 | 67.9 | 24.9 | 19.2 | 20 | 0.8 | 102 | 31 | -71 | 6 | 0 | -6 |
| LI | ETI | CFP 6 | M | F508del | F508del | 32.6 | 42.8 | 48.8 | 6 | 20.8 | 20.8 | 0 | 100 | N.D | N.D | 4 | 0 | -4 |
| LI | ETI | CFP 7 | F | F508del | F508del | 31.6 | 40.6 | 46.7 | 6.1 | 20.2 | 20.6 | 0.4 | 101 | 51 | -50 | 3 | 0 | -3 |
| LI | ETI | CFP 8 | F | F508del | F508del | 22.5 | 29.7 | 35.9 | 6.2 | 18.6 | 23.1 | 4.6 | 47 | 46 | -1 | 1 | 0 | -1 |
| None | TI | CFP 9^A^ | F | F508del | 2657+5G>A | 50.4 | 24.9 | 20.7 | -4.2 | 36.5 | 36.2 | -0.3 | 90 | 89 | -1 | 6 | 3 | -3 |
| TI | ETI | CFP 9 |  |  |  | 50.9 | 23.3 | 20.7 | -2.5 | 36.4 | 34.4 | -2 | 89 | 77 | -12 | 5 | 4 | -1 |
| None | TI | CFP 10^A^ | M | F508del | F508del | 21.7 | 39.8 | 42.6 | 2.9 | 22.4 | 21.4 | -1 | N.D | N.D | N.D | 2 | 5 | 3 |
| TI | ETI | CFP 10 |  |  |  | 22.6 | 40.7 | 45 | 4.3 | 21.4 | 23.4 | 2 | 99 | 43 | -56 | 5 | 0 | -5 |
| LI | TI | CFP 11 | M | F508del | F508del | 26.2 | 39.3 | 41.7 | 2.5 | 37 | 36.6 | -0.4 | 85 | N.D | N.D | 1 | 0 | -1 |

^A^CFP 9 and CFP 10 started the study on TI and later transitioned to ETI. Either no sweat chloride data at baseline or no matched post measurements were available for four patients (CFP 4, CFP 6, CFP 10, CFSP 11).

Abbreviations: No. – Number, FEV1pp – Forced expiratory volume in one second percent predicted, BMI - body mass index, ETI – Elexacaftor-Tezacaftor-Ivacaftor, TI – Tezacaftor-Ivacaftor, LI – Lumacaftor-Ivacaftor N.D – No data available.

**Supplementary Table 4. Annotations of all 37 genes commonly mutated in over 42% of lineages after CFTR modulation**

| **Number of lineages with at least 1 mutation in the gene post CFTR modulation** | **PGD Gene ID** | **Locus Tag** | **Gene Name** | **Product Name** | **Length (nucleotides)** | **PseudoCAP functional classes** | **Gene Length** | **Pathoadaptive** | **Antibiotic Resistance** |
| --- | --- | --- | --- | --- | --- | --- | --- | --- | --- |
| 9 | PGD106528 | PA1874 |  | hypothetical protein | 7407 | Antibiotic resistance and susceptibility | > 5000 bp | 1 | 0 |
| 9 | PGD112098 | PA4625 | cdrA | cyclic diguanylate-regulated TPS partner A, CdrA | 6465 | Secreted Factors (toxins, enzymes, alginate) // Cell wall / LPS / capsule | > 5000 bp | 0 | 0 |
| 8 | PGD107702 | PA2462 |  | hypothetical protein | 16884 | Hypothetical, unclassified, unknown | > 5000 bp | 0 | 0 |
| 8 | PGD110115 | PA3648 | opr86 | outer membrane protein Opr86 | 2394 | Transport of small molecules // Membrane proteins | 2000-5000 bp | 1 | 0 |
| 7 | PGD104938 | PA1086 | flgK | flagellar hook-associated protein 1 FlgK | 2052 | Cell wall / LPS / capsule // Motility & Attachment | 2000-5000 bp | 0 | 0 |
| 7 | PGD105056 | PA1144 |  | probable major facilitator superfamily (MFS) transporter | 1335 | Membrane proteins // Transport of small molecules | 1000-2000 bp | 0 | 0 |
| 7 | PGD107626 | PA2424 | pvdL | PvdL | 13029 | Adaptation, Protection | > 5000 bp | 0 | 0 |
| 7 | PGD109909 | PA3545 | algG | alginate-c5-mannuronan-epimerase AlgG | 1632 | Cell wall / LPS / capsule // Adaptation, Protection // Secreted Factors (toxins, enzymes, alginate) | 1000-2000 bp | 1 | 0 |
| 7 | PGD111922 | PA4541 | lepA | Pseudomonas aeruginosa-derived large extracellular protease, LepA | 4254 | Membrane proteins // Secreted Factors (toxins, enzymes, alginate) | 2000-5000 bp | 1 | 0 |
| 7 | PGD112036 | PA4594 |  | probable ATP-binding component of ABC transporter | 687 | Transport of small molecules | < 1000 bp | 1 | 0 |
| 7 | PGD112786 | PA4958 | fimW | FimW | 1823 | Hypothetical, unclassified, unknown | 1000-2000 bp | 0 | 0 |
| 6 | PGD102881 | PA0074 | ppkA | serine/threonine protein kinase PpkA | 3099 | Adaptation, Protection // Translation, post-translational modification, degradation // Protein secretion/export apparatus | 2000-5000 bp | 0 | 0 |
| 6 | PGD103771 | PA0517 | nirC | probable c-type cytochrome precursor | 360 | Biosynthesis of cofactors, prosthetic groups and carriers // Energy metabolism | < 1000 bp | 0 | 0 |
| 6 | PGD104021 | PA0641 |  | probable bacteriophage protein | 3615 | Related to phage, transposon, or plasmid | 2000-5000 bp | 0 | 0 |
| 6 | PGD104276 | PA0763 | mucA | anti-sigma factor MucA | 585 | Cell wall / LPS / capsule // Transcriptional regulators | < 1000 bp | 1 | 0 |
| 6 | PGD104678 | PA0958 | oprD | Basic amino acid, basic peptide and imipenem outer membrane porin OprD precursor | 1332 | Transport of small molecules | 1000-2000 bp | 1 | 0 |
| 6 | PGD105742 | PA1487 |  | probable carbohydrate kinase | 1416 | Putative enzymes | 1000-2000 bp | 0 | 0 |
| 6 | PGD105834 | PA1532 | dnaX | DNA polymerase subunits gamma and tau | 2046 | DNA replication, recombination, modification and repair | 2000-5000 bp | 1 | 0 |
| 6 | PGD106022 | PA1626 |  | probable major facilitator superfamily (MFS) transporter | 1275 | Membrane proteins // Transport of small molecules | 1000-2000 bp | 0 | 0 |
| 6 | PGD106616 | PA1918 |  | hypothetical protein | 1401 | Putative enzymes | 1000-2000 bp | 1 | 0 |
| 6 | PGD106910 | PA2065 | pcoA | copper resistance protein A precursor | 1899 | Adaptation, Protection | 1000-2000 bp | 1 | 0 |
| 6 | PGD107242 | PA2231 | pslA | PslA | 1437 | Cell wall / LPS / capsule | 1000-2000 bp | 1 | 0 |
| 6 | PGD107552 | PA2386 | pvdA | L-ornithine N5-oxygenase | 1332 | Adaptation, Protection | 1000-2000 bp | 0 | 0 |
| 6 | PGD107556 | PA2388 | fpvR | FpvR | 996 | Transport of small molecules // Membrane proteins // Transcriptional regulators | < 1000 bp | 0 | 0 |
| 6 | PGD107584 | PA2403 | fpvG | FpvG | 1212 | Membrane proteins | 1000-2000 bp | 0 | 0 |
| 6 | PGD107856 | PA2539 |  | conserved hypothetical protein | 1314 | Membrane proteins | 1000-2000 bp | 0 | 0 |
| 6 | PGD108926 | PA3064 | pelA | PelA | 2847 | Cell wall / LPS / capsule | 2000-5000 bp | 1 | 0 |
| 6 | PGD109014 | PA3105 | xcpQ | general secretion pathway protein D | 1977 | Protein secretion/export apparatus | 1000-2000 bp | 0 | 0 |
| 6 | PGD109475 | PA3329 |  | hypothetical protein | 1329 | Hypothetical, unclassified, unknown | 1000-2000 bp | 0 | 0 |
| 6 | PGD109647 | PA3414 |  | hypothetical protein | 585 | Hypothetical, unclassified, unknown | < 1000 bp | 0 | 0 |
| 6 | PGD109889 | PA3535 |  | probable serine protease | 2988 | Putative enzymes | 2000-5000 bp | 0 | 0 |
| 6 | PGD111272 | PA4226 | pchE | dihydroaeruginoic acid synthetase | 4317 | Secreted Factors (toxins, enzymes, alginate) // Transport of small molecules | 2000-5000 bp | 0 | 0 |
| 6 | PGD111360 | PA4270 | rpoB | DNA-directed RNA polymerase beta chain | 4074 | Transcription, RNA processing and degradation | 2000-5000 bp | 1 | 1 |
| 6 | PGD113042 | PA5086 | tli5b1 | type VI secretion lipase immunity protein, Tli5b1 | 642 | Hypothetical, unclassified, unknown | < 1000 bp | 0 | 0 |
| 6 | PGD113048 | PA5089 | tle5b | type VI secretion phospholipase D effector Tle5b | 2238 | Secreted Factors (toxins, enzymes, alginate) | 2000-5000 bp | 0 | 0 |
| 6 | PGD113098 | PA5114 |  | hypothetical protein | 3606 | Membrane proteins | 2000-5000 bp | 0 | 0 |
| 6 | PGD113946 | PA5531 | tonB1 | TonB1 | 1029 | Transport of small molecules | 1000-2000 bp | 0 | 0 |

**Supplementary Table 5. List of pathoadaptive genes used for enrichment analysis**

| **Locus_Tag** | **Gene_name** | **Product_name** | **Sources** |
| --- | --- | --- | --- |
| **PA0004** | ***gyrB*** | DNA gyrase subunit B | 12, 15 |
| **PA0011** | ***htrB1*** | acyltransferase HtrB1 | 12 |
| **PA0301** | ***spuE*** | polyamine transport protein | 17 |
| **PA0302** | ***spuF*** | polyamine transport protein PotG | 17 |
| **PA0313** |  | L-cysteine transporter of ABC system YecS | 12, 14 |
| **PA0319** |  | hypothetical protein | 15 |
| **PA0337** | ***ptsP*** | phosphoenolpyruvate-protein phosphotransferase PtsP | 17 |
| **PA0366** | ***laoC*** | LaoC | 14 |
| **PA0424** | ***mexR*** | multidrug resistance operon repressor MexR | 12 |
| **PA0425** | ***mexA*** | Resistance-Nodulation-Cell Division (RND) multidrug efflux membrane fusion protein MexA precursor | 12, 14, 16 |
| **PA0426** | ***mexB*** | Resistance-Nodulation-Cell Division (RND) multidrug efflux transporter MexB | 12, 15 |
| **PA0506** |  | probable acyl-CoA dehydrogenase | 14 |
| **PA0576** | ***rpoD*** | sigma factor RpoD | 15 |
| **PA0600** | ***agtS*** | two-component sensor, AgtS | 15 |
| **PA0652** | ***vfr*** | transcriptional regulator Vfr | 14 |
| **PA0705** | ***migA*** | alpha-1,6-rhamnosyltransferase MigA | 15 |
| **PA0707** | ***toxR*** | transcriptional regulator ToxR | 14 |
| **PA0762** | ***algU*** | sigma factor AlgU | 12, 15, 16, 17 |
| **PA0763** | ***mucA*** | anti-sigma factor MucA | 12, 16 |
| **PA0767** | ***lepA*** | GTP-binding protein LepA | 17 |
| **PA0788** |  | hypothetical protein | 13 |
| **PA0794** |  | probable aconitate hydratase | 13 |
| **PA0861** | ***rbdA*** | RbDA | 12, 15 |
| **PA0886** |  | probable C4-dicarboxylate transporter | 15 |
| **PA0904** | ***lysC*** | aspartate kinase alpha and beta chain | 15 |
| **PA0928** | ***gacS*** | sensor/response regulator hybrid | 13 |
| **PA0931** | ***pirA*** | ferric enterobactin receptor PirA | 15 |
| **PA0934** | ***relA*** | GTP pyrophosphokinase | 15, 17 |
| **PA0936** | ***lpxO2*** | lipopolysaccharide biosynthetic protein LpxO2 | 15 |
| **PA0958** | ***oprD*** | Basic amino acid, basic peptide and imipenem outer membrane porin OprD precursor | 12, 15, |
| **PA0977** |  | hypothetical protein | 12 |
| **PA0994** | ***cupC3*** | usher CupC3 | 13 |
| **PA0997** | ***pqsB*** | PqsB | 14 |
| **PA1097** | ***fleQ*** | transcriptional regulator FleQ | 14 |
| **PA1099** | ***fleR*** | two-component response regulator | 17 |
| **PA1115** |  | hypothetical protein | 15 |
| **PA1169** |  | probable lipoxygenase | 15 |
| **PA1179** | ***phoP*** | two-component response regulator PhoP | 14 |
| **PA1188** |  | hypothetical protein | 15 |
| **PA1259** | ***lhpH*** | LhpH | 15 |
| **PA1333** |  | hypothetical protein | 14 |
| **PA1430** | ***lasR*** | transcriptional regulator LasR | 12, 14, 16, 17 |
| **PA1471** |  | hypothetical protein | 12 |
| **PA1523** | ***xdhB*** | xanthine dehydrogenase | 13 |
| **PA1527** |  | conserved hypothetical protein | 15 |
| **PA1532** | ***dnaX*** | DNA polymerase subunits gamma and tau | 12 |
| **PA1544** | ***anr*** | transcriptional regulator Anr | 14 |
| **PA1567** |  | conserved hypothetical protein | 15 |
| **PA1580** | ***gltA*** | citrate synthase | 15 |
| **PA1613** |  | hypothetical protein | 13 |
| **PA1677** |  | conserved hypothetical protein | 12 |
| **PA1713** | ***exsA*** | transcriptional regulator ExsA | 14 |
| **PA1779** |  | assimilatory nitrate reductase | 15 |
| **PA1791** |  | hypothetical protein | 15 |
| **PA1843** | ***metH*** | methionine synthase | 15 |
| **PA1874** |  | hypothetical protein | 13 |
| **PA1918** |  | hypothetical protein | 15 |
| **PA1922** |  | probable TonB-dependent receptor | 15 |
| **PA1976** | ***ercS'*** | ErcS' | 15 |
| **PA2004** |  | conserved hypothetical protein | 15 |
| **PA2018** | ***mexY*** | Resistance-Nodulation-Cell Division (RND) multidrug efflux transporter MexY | 13, 15 |
| **PA2020** | ***mexZ*** | MexZ | 12, 14, 16 |
| **PA2057** | ***sppR*** | TonB-dependent receptor, SppR | 15 |
| **PA2065** | ***pcoA*** | copper resistance protein A precursor | 12 |
| **PA2099** |  | probable short-chain dehydrogenase | 12 |
| **PA2121** | ***bsrA*** | LysR-type transcriptional regulator, BsrA | 14 |
| **PA2164** |  | probable glycosyl hydrolase | 15 |
| **PA2231** | ***pslA*** | PslA | 13 |
| **PA2312** |  | probable transcriptional regulator | 14 |
| **PA2361** | ***icmF3*** | IcmF3 | 15 |
| **PA2426** | ***pvdS*** | sigma factor PvdS | 12 |
| **PA2435** |  | probable cation-transporting P-type ATPase | 14 |
| **PA2455** |  | hypothetical protein | 12 |
| **PA2490** |  | conserved hypothetical protein | 12 |
| **PA2491** | ***mexS*** | MexS | 12, 14, 16 |
| **PA2492** | ***mexT*** | transcriptional regulator MexT | 14 |
| **PA2543** |  | conserved hypothetical protein | 15 |
| **PA2586** | ***gacA*** | response regulator GacA | 17 |
| **PA2602** |  | 3-mercaptopropionate dioxygenase | 12 |
| **PA2647** | ***nuoL*** | NADH dehydrogenase I chain L | 17 |
| **PA2684** | ***tse5*** | Tse5 | 15 |
| **PA2771** |  | diguanylate cyclase with a self-blocked I-site, Dcsbis | 15 |
| **PA3059** | ***pelF*** | PelF | 17 |
| **PA3064** | ***pelA*** | PelA | 12, 15, 17 |
| **PA3141** | ***wbpM*** | nucleotide sugar epimerase/dehydratase WbpM | 12 |
| **PA3168** | ***gyrA*** | DNA gyrase subunit A | 12, 15 |
| **PA3192** | ***gltR*** | two-component response regulator GltR | 17 |
| **PA3217** | ***cyaB*** | CyaB | 14 |
| **PA3222** |  | hypothetical protein | 12 |
| **PA3271** |  | probable two-component sensor | 13, 15 |
| **PA3290** | ***tle1*** | Tle1 | 12 |
| **PA3346** | ***hsbR*** | HptB-dependent secretion and biofilm regulator HsbR | 15 |
| **PA3458** |  | probable transcriptional regulator | 12 |
| **PA3476** | ***rhlI*** | autoinducer synthesis protein RhlI | 14 |
| **PA3477** | ***rhlR*** | transcriptional regulator RhlR | 14, 17 |
| **PA3545** | ***algG*** | alginate-c5-mannuronan-epimerase AlgG | 17 |
| **PA3565** |  | probable transcriptional regulator | 14 |
| **PA3574** | ***nalD*** | NalD | 12, 14 |
| **PA3620** | ***mutS*** | DNA mismatch repair protein MutS | 15 |
| **PA3648** | ***opr86*** | outer membrane protein Opr86 | 15 |
| **PA3690** |  | probable metal-transporting P-type ATPase | 15 |
| **PA3703** | ***wspF*** | probable methylesterase | 14 |
| **PA3704** | ***wspE*** | probable chemotaxis sensor/effector fusion protein | 12 |
| **PA3708** | ***wspA*** | probable chemotaxis transducer | 12 |
| **PA3817** |  | probable methyltransferase | 14 |
| **PA3878** | ***narX*** | two-component sensor NarX | 15 |
| **PA3895** |  | probable transcriptional regulator | 13 |
| **PA3939** |  | hypothetical protein | 12 |
| **PA4020** | ***mpl*** | UDP-N-acetylmuramate:L-alanyl-gamma-D-glutamyl-meso-diaminopimelate ligase | 12 |
| **PA4071** |  | hypothetical protein | 15 |
| **PA4082** | ***cupB5*** | adhesive protein CupB5 | 15 |
| **PA4109** | ***ampR*** | transcriptional regulator AmpR | 15 |
| **PA4110** | ***ampC*** | beta-lactamase precursor | 13, 15 |
| **PA4112** |  | probable sensor/response regulator hybrid | 15 |
| **PA4211** | ***phzB1*** | probable phenazine biosynthesis protein | 12 |
| **PA4221** | ***fptA*** | Fe(III)-pyochelin outer membrane receptor precursor | 15 |
| **PA4266** | ***fusA1*** | elongation factor G | 13, 15 |
| **PA4269** | ***rpoC*** | DNA-directed RNA polymerase beta* chain | 15 |
| **PA4270** | ***rpoB*** | DNA-directed RNA polymerase beta chain | 15 |
| **PA4293** | ***pprA*** | two-component sensor PprA | 15 |
| **PA4311** |  | conserved hypothetical protein | 12 |
| **PA4367** | ***bifA*** | BifA | 12 |
| **PA4391** |  | hypothetical protein | 15, 17 |
| **PA4418** | ***ftsI*** | penicillin-binding protein 3 | 13, 15 |
| **PA4420** |  | conserved hypothetical protein | 14 |
| **PA4462** | ***rpoN*** | RNA polymerase sigma-54 factor | 14 |
| **PA4489** | ***magD*** | MagD | 13 |
| **PA4513** |  | probable oxidoreductase | 13 |
| **PA4522** | ***ampD*** | beta-lactamase expression regulator AmpD | 14 |
| **PA4526** | ***pilB*** | type 4 fimbrial biogenesis protein PilB | 14 |
| **PA4528** | ***pilD*** | type 4 prepilin peptidase PilD | 12 |
| **PA4541** | ***lepA*** | Pseudomonas aeruginosa-derived large extracellular protease, LepA | 15 |
| **PA4594** |  | probable ATP-binding component of ABC transporter | 15 |
| **PA4600** | ***nfxB*** | transcriptional regulator NfxB | 12 |
| **PA4601** | ***morA*** | motility regulator | 12 |
| **PA4642** |  | hypothetical protein | 12 |
| **PA4661** | ***pagL*** | Lipid A 3-O-deacylase | 17 |
| **PA4689** |  | hypothetical protein | 15 |
| **PA4777** | ***pmrB*** | PmrB: two-component regulator system signal sensor kinase PmrB | 15 |
| **PA4796** |  | hypothetical protein | 14 |
| **PA4848** | ***accC*** | biotin carboxylase | 14 |
| **PA4856** | ***retS*** | RetS (Regulator of Exopolysaccharide and Type III Secretion) | 12 |
| **PA4918** | ***pcnA*** | nicotinamidase, PcnA | 15 |
| **PA4963** |  | hypothetical protein | 12 |
| **PA5015** | ***aceE*** | pyruvate dehydrogenase | 12, 13 |
| **PA5016** | ***aceF*** | dihydrolipoamide acetyltransferase | 12 |
| **PA5040** | ***pilQ*** | Type 4 fimbrial biogenesis outer membrane protein PilQ precursor | 12 |
| **PA5048** |  | probable nuclease | 17 |
| **PA5060** | ***phaF*** | polyhydroxyalkanoate synthesis protein PhaF | 12 |
| **PA5160** |  | drug efflux transporter | 15 |
| **PA5177** |  | probable hydrolase | 12 |
| **PA5213** | ***gcvP1*** | glycine cleavage system protein P1 | 13 |
| **PA5238** |  | probable O-antigen acetylase | 15 |
| **PA5266** | ***vgrG6*** | VgrG6 | 12 |
| **PA5291** | ***betT2*** | BetT2 | 12 |
| **PA5338** | ***spoT*** | guanosine-3',5'-bis(diphosphate) 3'-pyrophosphohydrolase | 17 |
| **PA5418** | ***soxA*** | sarcosine oxidase alpha subunit | 13 |
| **PA5437** |  | probable transcriptional regulator | 12 |
| **PA5516** | ***pdxY*** | pyridoxamine kinase | 12 |

Note: Study numbers refer to references in supplementary and not the main manuscript.

**Supplementary Table 6. List of antibiotic resistant genes used for enrichment analysis**

| **Locus_tag** | **Gene** | **Product_name** | **Sources** |
| --- | --- | --- | --- |
| **PA0004** | ***gyrB*** | DNA gyrase subunit B | 18, 19, 20 |
| **PA0005** | ***lptA*** | lysophosphatidic acid acyltransferase, LptA | 18, 19, 20 |
| **PA0018** | ***fmt*** | methionyl-tRNA formyltransferase | 18, 19, 20 |
| **PA0058** | ***dsbM*** | DsbM | 18, 19, 20 |
| **PA0156** | **-** | Resistance-Nodulation-Cell Division (RND) triclosan efflux membrane fusion protein, TriA | 21 |
| **PA0157** | **-** | Resistance-Nodulation-Cell Division (RND) triclosan efflux membrane fusion protein, TriB | 21 |
| **PA0158** | **-** | Resistance-Nodulation-Cell Division (RND) triclosan efflux transporter, TriC | 21 |
| **PA0301** | ***spuE*** | polyamine transport protein | 18, 19, 20 |
| **PA0302** | ***spuF*** | polyamine transport protein PotG | 18, 19, 20 |
| **PA0355** | ***pfpI*** | protease PfpI | 18, 19, 20 |
| **PA0392** | ***yggT*** | conserved hypothetical protein | 18, 19, 20 |
| **PA0402** | ***pyrB*** | aspartate carbamoyltransferase | 18, 19, 20 |
| **PA0424** | ***mexR*** | multidrug resistance operon repressor MexR | 18, 19, 20, 21 |
| **PA0425** | ***mexA*** | Resistance-Nodulation-Cell Division (RND) multidrug efflux membrane fusion protein MexA precursor | 18, 19, 20, 21 |
| **PA0426** | ***mexB*** | Resistance-Nodulation-Cell Division (RND) multidrug efflux transporter MexB | 18, 19, 20, 21 |
| **PA0427** | ***oprM*** | Major intrinsic multiple antibiotic resistance efflux outer membrane protein OprM precursor | 18, 19, 20, 21 |
| **PA0463** | ***creB*** | two-component response regulator CreB | 18, 19, 20 |
| **PA0464** | ***creC*** | two-component sensor CreC | 18, 19, 20 |
| **PA0465** | ***creD*** | inner membrane protein CreD | 18, 19, 20 |
| **PA0486** | ***yihE*** | conserved hypothetical protein | 18, 19, 20 |
| **PA0487** | ***modR*** | probable molybdenum transport regulator | 18, 19, 20 |
| **PA0610** | ***prtN*** | transcriptional regulator PrtN | 18, 19, 20 |
| **PA0611** | ***prtR*** | transcriptional regulator PrtR | 18, 19, 20 |
| **PA0612** | ***ptrB*** | repressor, PtrB | 18, 19, 20 |
| **PA0668.4** | **-** | 23S ribosomal RNA | 20 |
| **PA0706** | **-** | chloramphenicol acetyltransferase | 21 |
| **PA0749** | **-** | hypothetical protein | 21 |
| **PA0779** | ***asrA*** | AsrA | 18, 19, 20 |
| **PA0807** | ***ampDh3*** | AmpDh3 | 18, 19, 20 |
| **PA0869** | **PBP6/7** | D-alanyl-D-alanine-endopeptidase | 18, 19, 20 |
| **PA0893** | ***argR*** | transcriptional regulator ArgR | 18, 19, 20 |
| **PA0905** | **-** | RsmA | 21 |
| **PA0958** | ***oprD*** | Basic amino acid, basic peptide and imipenem outer membrane porin OprD precursor | 18, 19, 20 |
| **PA1129** | **-** | fosfomycin resistance protein, FosA | 21 |
| **PA1178** | ***oprH*** | PhoP/Q and low Mg2+ inducible outer membrane protein H1 precursor | 18, 19, 20 |
| **PA1179** | ***phoP*** | two-component response regulator PhoP | 18, 19, 20 |
| **PA1180** | ***phoQ*** | two-component sensor PhoQ | 18, 19, 20 |
| **PA1343** | ***pagP*** | hypothetical protein | 18, 19, 20 |
| **PA1345** | ***gshB*** | hypothetical protein | 18, 19, 20 |
| **PA1361** | **-** | NorM | 21 |
| **PA1375** | ***pdxB*** | erythronate-4-phosphate dehydrogenase | 18, 19, 20 |
| **PA1409** | ***aphA*** | acetylpolyamine aminohydrolase | 18, 19, 20 |
| **PA1430** | ***lasR*** | transcriptional regulator LasR | 18, 19, 20 |
| **PA1435** | **-** | probable Resistance-Nodulation-Cell Division (RND) efflux membrane fusion protein precursor | 21 |
| **PA1436** | **-** | probable Resistance-Nodulation-Cell Division (RND) efflux transporter | 21 |
| **PA1588** | ***sucC*** | succinyl-CoA synthetase beta chain | 18, 19, 20 |
| **PA1589** | ***sucD*** | succinyl-CoA synthetase alpha chain | 18, 19, 20 |
| **PA1777** | ***oprF*** | Major porin and structural outer membrane porin OprF precursor | 18, 19, 20 |
| **PA1796** | ***folD*** | 5,10-methylene-tetrahydrofolate dehydrogenase / cyclohydrolase | 18, 19, 20 |
| **PA1797** | ***-*** | hypothetical protein | 18, 19, 20 |
| **PA1798** | ***parS*** | two-component sensor, ParS | 18, 19, 20, 21 |
| **PA1799** | ***parR*** | two-component response regulator, ParR | 18, 19, 20, 21 |
| **PA1801** | ***clpP*** | ClpP | 18, 19, 20 |
| **PA1803** | ***lon*** | Lon protease | 18, 19, 20 |
| **PA1812** | ***mltD*** | membrane-bound lytic murein transglycosylase D precursor | 18, 19, 20 |
| **PA1886** | ***polB*** | DNA polymerase II | 18, 19, 20 |
| **PA2006** | **-** | probable major facilitator superfamily (MFS) transporter | 18, 19, 20 |
| **PA2018** | ***mexY*** | Resistance-Nodulation-Cell Division (RND) multidrug efflux transporter MexY | 18, 19, 20, 21 |
| **PA2019** | ***mexX*** | Resistance-Nodulation-Cell Division (RND) multidrug efflux membrane fusion protein MexX precursor | 18, 19, 20 |
| **PA2020** | ***mexZ*** | MexZ | 18, 19, 20, 21 |
| **PA2023** | ***galU*** | UTP--glucose-1-phosphate uridylyltransferase | 18, 19, 20 |
| **PA2050** | **-** | probable sigma-70 factor, ECF subfamily | 18, 19, 20 |
| **PA2071** | ***fusA2*** | elongation factor G | 18, 19, 20 |
| **PA2227** | ***vqsM*** | AraC-type transcriptional regulator VqsM | 18, 19, 20 |
| **PA2272** | **PBP3a** | penicillin-binding protein 3A | 18, 19, 20 |
| **PA2273** | ***soxR*** | SoxR | 18, 19, 20, 21 |
| **PA2489** | **-** | probable transcriptional regulator | 18, 19, 20 |
| **PA2490** | ***ydbB*** | conserved hypothetical protein | 18, 19, 20 |
| **PA2491** | ***mexS*** | MexS | 18, 19, 20, 21 |
| **PA2492** | ***mexT*** | transcriptional regulator MexT | 18, 19, 20 |
| **PA2493** | ***mexE*** | Resistance-Nodulation-Cell Division (RND) multidrug efflux membrane fusion protein MexE precursor | 18, 19, 20, 21 |
| **PA2494** | ***mexF*** | Resistance-Nodulation-Cell Division (RND) multidrug efflux transporter MexF | 18, 19, 20, 21 |
| **PA2495** | ***oprN*** | Multidrug efflux outer membrane protein OprN precursor | 18, 19, 20, 21 |
| **PA2522** | ***czcC*** | outer membrane protein precursor CzcC | 18, 19, 20 |
| **PA2523** | ***czcR*** | CzcR | 18, 19, 20 |
| **PA2524** | ***czcS*** | CzcS | 18, 19, 20 |
| **PA2525** | ***opmB*** | OpmB | 18, 19, 20, 21 |
| **PA2526** | ***muxC*** | MuxC | 18, 19, 20, 21 |
| **PA2527** | ***muxB*** | MuxB | 18, 19, 20, 21 |
| **PA2528** | ***muxA*** | MuxA | 18, 19, 20, 21 |
| **PA2615** | ***ftsK*** | cell division protein FtsK | 18, 19, 20 |
| **PA2621** | ***clpS*** | ClpS | 18, 19, 20 |
| **PA2642** | ***nuoG*** | NADH dehydrogenase I chain G | 18, 19, 20 |
| **PA2649** | ***nuoN*** | NADH dehydrogenase I chain N | 18, 19, 20 |
| **PA2797** | **-** | hypothetical protein | 18, 19, 20 |
| **PA2798** | **-** | probable two-component response regulator | 18, 19, 20 |
| **PA2809** | ***copR*** | two-component response regulator, CopR | 18, 19, 20 |
| **PA2810** | ***copS*** | two-component sensor, CopS | 18, 19, 20 |
| **PA2830** | ***htpX*** | heat shock protein HtpX | 18, 19, 20 |
| **PA3005** | ***nagZ*** | beta-N-acetyl-D-glucosaminidase | 18, 19, 20 |
| **PA3013** | ***foaB*** | fatty-acid oxidation complex beta-subunit | 18, 19, 20 |
| **PA3014** | ***faoA*** | fatty-acid oxidation complex alpha-subunit | 18, 19, 20 |
| **PA3047** | **PBP4** | probable D-alanyl-D-alanine carboxypeptidase | 18, 19, 20 |
| **PA3050** | ***pyrD*** | dihydroorotate dehydrogenase | 18, 19, 20 |
| **PA3077** | ***cprR*** | CprR | 18, 19, 20, 21 |
| **PA3078** | ***cprS*** | CprS | 18, 19, 20, 21 |
| **PA3141** | ***capD*** | nucleotide sugar epimerase/dehydratase WbpM | 18, 19, 20 |
| **PA3168** | ***gyrA*** | DNA gyrase subunit A | 18, 19, 20 |
| **PA3204** | **-** | two-component response regulator CpxR | 21 |
| **PA3521** | ***opmE*** | OpmE | 18, 19, 20, 21 |
| **PA3522** | ***mexQ*** | MexQ | 18, 19, 20, 21 |
| **PA3523** | ***mexP*** | MexP | 18, 19, 20, 21 |
| **PA3533** | ***grxD*** | GrxD | 18, 19, 20 |
| **PA3554** | **-** | ArnA | 21 |
| **PA3574** | ***nalD*** | NalD | 18, 19, 20, 21 |
| **PA3602** | ***yerD*** | conserved hypothetical protein | 18, 19, 20 |
| **PA3676** | ***mexK*** | MexK | 18, 19, 20, 21 |
| **PA3677** | ***mexJ*** | MexJ | 18, 19, 20, 21 |
| **PA3678** | ***mexL*** | MexL | 18, 19, 20, 21 |
| **PA3719** | ***armR*** | antirepressor for MexR, ArmR | 18, 19, 20, 21 |
| **PA3721** | ***nalC*** | NalC | 18, 19, 20, 21 |
| **PA3822** | **-** | conserved hypothetical protein | 21 |
| **PA3999** | **PBP5** | D-ala-D-ala-carboxypeptidase | 18, 19, 20 |
| **PA4001** | ***sltB1*** | soluble lytic transglycosylase B | 18, 19, 20 |
| **PA4003** | **PBP2** | penicillin-binding protein 2 | 18, 19, 20 |
| **PA4020** | ***mpl*** | UDP-N-acetylmuramate:L-alanyl-gamma-D-glutamyl-meso-diaminopimelate ligase | 18, 19, 20 |
| **PA4069** | **-** | hypothetical protein | 18, 19, 20 |
| **PA4109** | ***ampR*** | transcriptional regulator AmpR | 18, 19, 20 |
| **PA4110** | ***ampC*** | beta-lactamase precursor | 18, 19, 20, 21 |
| **PA4119** | ***aph*** | aminoglycoside 3'-phosphotransferase type IIb | 18, 19, 20, 21 |
| **PA4136** | **-** | probable major facilitator superfamily (MFS) transporter | 21 |
| **PA4205** | ***mexG*** | hypothetical protein | 18, 19, 20, 21 |
| **PA4206** | ***mexH*** | probable Resistance-Nodulation-Cell Division (RND) efflux membrane fusion protein precursor | 18, 19, 20, 21 |
| **PA4207** | ***mexI*** | probable Resistance-Nodulation-Cell Division (RND) efflux transporter | 18, 19, 20, 21 |
| **PA4208** | ***opmD*** | probable outer membrane protein precursor | 18, 19, 20, 21 |
| **PA4218** | ***ampP*** | AmpP | 18, 19, 20 |
| **PA4238** | ***rpoA*** | DNA-directed RNA polymerase alpha chain | 18, 19, 20 |
| **PA4260** | ***rplB*** | 50S ribosomal protein L2 | 18, 19, 20 |
| **PA4266** | ***fusA1*** | elongation factor G | 18, 19, 20 |
| **PA4269** | ***rpoC*** | DNA-directed RNA polymerase beta* chain | 18, 19, 20 |
| **PA4270** | ***rpoB*** | DNA-directed RNA polymerase beta chain | 18, 19, 20 |
| **PA4273** | ***rplA*** | 50S ribosomal protein L1 | 18, 19, 20 |
| **PA4280.2** | **-** | 23S ribosomal RNA | 20 |
| **PA4315** | ***mvaT*** | transcriptional regulator MvaT, P16 subunit | 18, 19, 20 |
| **PA4374** | ***mexV*** | Resistance-Nodulation-Cell Division (RND) multidrug efflux membrane fusion protein MexV | 18, 19, 20, 21 |
| **PA4375** | ***mexW*** | Resistance-Nodulation-Cell Division (RND) multidrug efflux transporter MexW | 18, 19, 20, 21 |
| **PA4380** | ***colS*** | two-component sensor ColS | 18, 19, 20 |
| **PA4381** | ***colR*** | two-component response regulator ColR | 18, 19, 20 |
| **PA4393** | ***ampG*** | AmpG | 18, 19, 20 |
| **PA4406** | ***lpxC*** | UDP-3-O-acyl-N-acetylglucosamine deacetylase | 18, 19, 20 |
| **PA4418** | **PBP3** | penicillin-binding protein 3 | 18, 19, 20 |
| **PA4444** | ***mltB1*** | soluble and membrane-bound lytic transglycosylases | 18, 19, 20 |
| **PA4462** | ***rpoN*** | RNA polymerase sigma-54 factor | 18, 19, 20 |
| **PA4521** | ***ampE*** | AmpE | 18, 19, 20 |
| **PA4522** | ***ampD*** | beta-lactamase expression regulator AmpD | 18, 19, 20 |
| **PA4567** | ***rpmA*** | 50S ribosomal protein L27 | 18, 19, 20 |
| **PA4568** | ***rplU*** | 50S ribosomal protein L21 | 18, 19, 20 |
| **PA4597** | ***oprJ*** | Multidrug efflux outer membrane protein OprJ precursor | 18, 19, 20, 21 |
| **PA4598** | ***mexD*** | Resistance-Nodulation-Cell Division (RND) multidrug efflux transporter MexD | 18, 19, 20, 21 |
| **PA4599** | ***mexC*** | Resistance-Nodulation-Cell Division (RND) multidrug efflux membrane fusion protein MexC precursor | 18, 19, 20, 21 |
| **PA4600** | ***nfxB*** | transcriptional regulator NfxB | 18, 19, 20, 21 |
| **PA4661** | ***pagL*** | Lipid A 3-O-deacylase | 18, 19, 20 |
| **PA4671** | ***rplY*** | probable ribosomal protein L25 | 18, 19, 20 |
| **PA4690.2** | **-** | 23S ribosomal RNA | 20 |
| **PA4700** | **PBP1b** | penicillin-binding protein 1B | 18, 19, 20 |
| **PA4748** | ***tpiA*** | triosephosphate isomerase | 18, 19, 20 |
| **PA4751** | ***ftsH*** | cell division protein FtsH | 18, 19, 20 |
| **PA4773** | **-** | SpeD2 | 18, 19, 20 |
| **PA4774** | **-** | SpeE2 | 18, 19, 20 |
| **PA4775** | **-** | hypothetical protein | 18, 19, 20 |
| **PA4776** | ***pmrA*** | PmrA: two-component regulator system response regulator PmrA | 18, 19, 20 |
| **PA4777** | ***pmrB*** | PmrB: two-component regulator system signal sensor kinase PmrB | 18, 19, 20, 21 |
| **PA4878** | ***brlR*** | BrlR | 18, 19, 20 |
| **PA4944** | ***hfq*** | Hfq | 18, 19, 20 |
| **PA4964** | ***parC*** | topoisomerase IV subunit A | 18, 19, 20 |
| **PA4967** | ***parE*** | topoisomerase IV subunit B | 18, 19, 20 |
| **PA4974** | **-** | probable outer membrane protein precursor | 21 |
| **PA4990** | **-** | SMR multidrug efflux transporter | 21 |
| **PA5000** | ***wapR*** | alpha-1,3-rhamnosyltransferase WapR | 18, 19, 20 |
| **PA5038** | ***aroB*** | 3-dehydroquinate synthase | 18, 19, 20 |
| **PA5045** | **PBP1a** | penicillin-binding protein 1A | 18, 19, 20 |
| **PA5117** | ***typA*** | regulatory protein TypA | 18, 19, 20 |
| **PA5199** | ***amgS*** | AmgS | 18, 19, 20 |
| **PA5200** | ***amgR*** | AmgR | 18, 19, 20 |
| **PA5235** | ***glpT*** | glycerol-3-phosphate transporter | 18, 19, 20 |
| **PA5297** | ***poxB*** | pyruvate dehydrogenase (cytochrome) | 18, 19, 20 |
| **PA5332** | ***crc*** | catabolite repression control protein | 18, 19, 20 |
| **PA5366** | ***pstB*** | ATP-binding component of ABC phosphate transporter | 18, 19, 20 |
| **PA5369.2** | **-** | 23S ribosomal RNA | 20 |
| **PA5471** | ***armZ*** | ArmZ | 18, 19, 20 |
| **PA5471.1** | **-** | PA5471 leader peptide | 18, 19, 20 |
| **PA5485** | ***ampDh2*** | AmpDh2 | 18, 19, 20 |
| **PA5514** | **-** | probable beta-lactamase | 21 |
| **PA5528** | **-** | hypothetical protein | 18, 19, 20 |
| **PA5542** | **-** | Pseudomonas imipenem beta-lactamase PIB-1 | 18, 19, 20 |

Note: Study numbers refer to references in supplementary and not the main manuscript.

**Supplementary Table 7. Number of non-synonymous, indel or frameshift mutations in *mutS, mutL* and *uvrD* in all lineages**

| **Patient code_multi-locus sequence type** | **Number of non-synonomous (missense & nonsense), modifier indels (insertions & deletions) or frameshift mutations in hypermutator genes** | | | |
| --- | --- | --- | --- | --- |
|  | ***mutS*** | ***mutL*** | ***uvrD*** | **Sum** |
| **CFP1_801** | 1 | 0 | 2 | 3 |
| **CFP1_775** | 1 | 0 | 3 | 4 |
| **CFP2_822** | 1 | 0 | 0 | 1 |
| **CFP3_801** | 1 | 0 | 2 | 3 |
| **CFP4_775** | 4 | 0 | 5 | 9 |
| **CFP5_262** | 0 | 1 | 0 | 1 |
| **CFP6_260** | 1 | 0 | 2 | 3 |
| **CFP7_262** | 0 | 0 | 0 | 0 |
| **CFP7_801** | 0 | 0 | 2 | 2 |
| **CFP8_829** | 1 | 2 | 3 | 6 |
| **CFP9_845** | 2 | 2 | 2 | 6 |
| **CFP9_801** | 0 | 1 | 2 | 3 |
| **CFP10_1637** | 1 | 0 | 2 | 3 |
| **CFP11_801** | 0 | 0 | 2 | 2 |

**Supplementary References**

1. Bolger AM, Lohse M, Usadel B. Trimmomatic: a flexible trimmer for Illumina sequence data. Bioinformatics **2014**; 30:2114-20.

2. Prjibelski A, Antipov D, Meleshko D, Lapidus A, Korobeynikov A. Using SPAdes de novo assembler. Curr Protoc Bioinformatics **2020**; 70:e102.

3. Seemann T. Prokka: rapid prokaryotic genome annotation. Bioinformatics **2014**; 30:2068-9.

4. Li H, Durbin R. Fast and accurate long-read alignment with Burrows-Wheeler transform. Bioinformatics **2010**; 26:589-95.

5. Parks DH, Imelfort M, Skennerton CT, Hugenholtz P, Tyson GW. CheckM: assessing the quality of microbial genomes recovered from isolates, single cells, and metagenomes. Genome Res **2015**; 25:1043-55.

6. Stover CK, Pham XQ, Erwin AL, et al. Complete genome sequence of *Pseudomonas aeruginosa* PAO1, an opportunistic pathogen. Nature **2000**; 406:959-64.

7. Eklöf J, Misiakou MA, Sivapalan P, et al. Persistence and genetic adaptation of *Pseudomonas aeruginosa* in patients with chronic obstructive pulmonary disease. Clin Microbiol Infect **2022**; 28:990-5.

8. Marvig RL, Sommer LM, Molin S, Johansen HK. Convergent evolution and adaptation of *Pseudomonas aeruginosa* within patients with cystic fibrosis. Nat Genet **2015**; 47:57-64.

9. Feliziani S, Marvig RL, Luján AM, et al. Coexistence and within-host evolution of diversified lineages of hypermutable *Pseudomonas aeruginosa* in long-term cystic fibrosis infections. PLoS Genet **2014**; 10:e1004651.

10. Smith EE, Buckley DG, Wu Z, et al. Genetic adaptation by *Pseudomonas aeruginosa* to the airways of cystic fibrosis patients. PNAS **2006**; 103:8487–92.

11. Marvig RL, Johansen HK, Molin S, Jelsbak L. Genome analysis of a transmissible lineage of *Pseudomonas aeruginosa* reveals pathoadaptive mutations and distinct evolutionary paths of hypermutators. PLOS Genetics **2013**; 9:e1003741.

12. Sommer LM, Alanin MC, Marvig RL, et al. Bacterial evolution in PCD and CF patients follows the same mutational steps. Sci Rep **2016**; 6:28732.

13. Fischer S, Klockgether J, Gonzalez Sorribes M, Dorda M, Wiehlmann L, Tümmler B. Sequence diversity of the *Pseudomonas aeruginosa* population in loci that undergo microevolution in cystic fibrosis airways. Access Microbiol **2021**; 3:000286.

14. López-Causapé C, Sommer LM, Cabot G, et al. Evolution of the *Pseudomonas aeruginosa* mutational resistome in an international Cystic Fibrosis clone. Sci Rep **2017**; 7:5555.

15. Colque CA, Albarracín Orio AG, Feliziani S, et al. Hypermutator *Pseudomonas aeruginosa* exploits multiple genetic pathways to develop multidrug resistance during long-term infections in the airways of cystic fibrosis patients. Antimicrob Agents Chemother **2020**; 64:e02142-19.

16. Cabot G, Zamorano L, Moyà B, et al. Evolution of *Pseudomonas aeruginosa* antimicrobial resistance and fitness under low and high mutation rates. Antimicrob Agents Chemother **2016**; 60:1767–78.

17. Alcock BP, Huynh W, Chalil R, et al. CARD 2023: expanded curation, support for machine learning, and resistome prediction at the Comprehensive Antibiotic Resistance Database. Nucleic Acids Res **2023**; 51:D690-d9.

18. Winsor GL, Griffiths EJ, Lo R, Dhillon BK, Shay JA, Brinkman FS. Enhanced annotations and features for comparing thousands of *Pseudomonas* genomes in the *Pseudomonas* genome database. Nucleic Acids Res **2016**; 44:D646-53.

19. Hall KM, Pursell ZF, Morici LA. The role of the *Pseudomonas aeruginosa* hypermutator phenotype on the shift from acute to chronic virulence during respiratory infection. Front Cell Infect Microbiol **2022**; 12:943346.

20. Oliver A, Cantón R, Campo P, Baquero F, Blázquez J. High frequency of hypermutable *Pseudomonas aeruginosa* in cystic fibrosis lung infection. Science **2000**; 288:1251.

21. Oliver A, Baquero F, Blázquez J. The mismatch repair system (*mutS, mutL* and *uvrD* genes) in *Pseudomonas aeruginosa*: molecular characterization of naturally occurring mutants. Mol Microbiol **2002**; 43:1641-50.

22. Hogardt M, Schubert S, Adler K, Götzfried M, Heesemann J. Sequence variability and functional analysis of *mutS* of hypermutable *Pseudomonas aeruginosa* cystic fibrosis isolates. Int J Med Microbiol **2006**; 296:313-20.

23. CLSI M100 ED33 - Performance Standards for Antimicrobial Susceptibilty Testing. Clinical and Laboratory Standards Institute (CLSI), USA **2023**.

**Other supplementary data**

**Supplementary data 2 (excel document). List of all mutations unique to strains isolated after CFTR modulation.** Each sheet has mutations from the corresponding cystic fibrosis participant (CFP)_multi-locus sequence type.

**Supplementary data 3 (excel document). List of all mutations in *mutS, mutL* and *uvrD* in all lineages.** Each sheet has mutations from the corresponding cystic fibrosis participant (CFP)_multi-locus sequence type.
